# A systematic review of the methodology of trade-off analysis in agriculture

**DOI:** 10.1038/s43016-024-00926-x

**Published:** 2024-03-05

**Authors:** Timo S. Breure, Natalia Estrada-Carmona, Athanasios Petsakos, Elisabetta Gotor, Boris Jansen, Jeroen C. J. Groot

**Affiliations:** 1 Farming Systems Ecology, Wageningen University and Research, Wageningen, The Netherlands; 2 Bioversity International, Montpellier, France; 3 Bioversity International, Rome, Italy; 4 Institute for Biodiversity and Ecosystem Dynamics, University of Amsterdam, Amsterdam, The Netherlands

**Keywords:** Environmental impact, Agroecology, Ecosystem services, Sustainability, Ecological modelling

## Abstract

Trade-off analysis (TOA) is central to policy and decision-making aimed at promoting sustainable agricultural landscapes. Yet, a generic methodological framework to assess trade-offs in agriculture is absent, largely due to the wide range of research disciplines and objectives for which TOA is used. In this study, we systematically reviewed 119 studies that have implemented TOAs in landscapes and regions dominated by agricultural systems around the world. Our results highlight that TOAs tend to be unbalanced, with a strong emphasis on productivity rather than environmental and socio-cultural services. TOAs have mostly been performed at farm or regional scales, rarely considering multiple spatial scales simultaneously. Mostly, TOAs fail to include stakeholders at study development stage, disregard recommendation uncertainty due to outcome variability and overlook risks associated with the TOA outcomes. Increased attention to these aspects is critical for TOAs to guide agricultural landscapes towards sustainability.

## Abstract (web summary)

Trade-off analyses are common in agriculture, but differences in their design and application make comparisons difficult. This systematic review examines the existing trade-off analysis literature in terms of methods, spatial scale, farming system, indicators and other analytical features.

## Main

Contemporary agriculture should not only provide food, fibre, feed and fuel but also environmental and socio-economic benefits for rural communities and beyond^1^. To ensure that agriculture delivers multiple services while minimizing its negative impacts, society must be aware of the trade-offs and synergies that may arise. The nature of these trade-offs depends on location-specific natural, social and cultural conditions that place constraints on the inputs and outputs of an agricultural system. For example, market-based farmers are concerned with enhancing commodity production, whereas the priority of subsistence farmers lies with improving food security^2^. The global imperative to achieve the United Nations Sustainable Development Goals (SDGs) underscores the need to reduce the environmental impact of land use practices and strengthen equitable social outcomes at both landscape and community levels. However, achieving the SDGs might require sacrifices to primary productivity and economic revenues. Thus, to reconcile the demands of agriculture and inform decision-making, an analysis is required of potential trade-offs measured against agronomic, environmental, economic and social indicators^3^.

Trade-off analysis (TOA) was established as a concept to generate quantitative information on competing (trade-offs) or complementary (synergies) indicators that can be used to guide policy and decision-making^4^. A typical TOA project starts with three preparatory steps: formulation of the research question, identification of which indicators to assess, and formulation of hypotheses about the relationships between the indicators and the associated trade-offs and synergies. Subsequently, the management, policy or technological changes that affect the TOA indicators can be identified and included in the analysis framework. Then, the trade-offs and synergies under changing conditions or scenarios can be quantified and, finally, the results are communicated to relevant stakeholders to inform decision-making and policy^4^. Since its first implementation in the context of agriculture, a wide range of methods have been used to conduct TOAs, including optimization, simulations, qualitative, econometric and narrative-based approaches. In some cases, these approaches are deployed in a spatially explicit manner with the support of geographic information systems (GIS)^5^.

Although important advances have been made regarding TOA in agricultural contexts, researchers have expressed concerns about the scope and methodological limitations of published studies. These concerns relate to the limited transfer of the academic knowledge generated by TOA into decision- and policy-making due to the inability to take into account social and cultural factors^6^, the sparsity of multi- and cross-scale assessments^3,5–7^, and the limited representation of uncertainty^8,9^ and risk analysis^5^.

The concerns reported in the literature on the limitations of TOA analysis can indeed have important implications. First, failure to recognize the importance of scale (spatial, temporal, jurisdictional and legislative) in TOA may lead to erroneous inferences on how the relationships between trade-offs and indicators develop across scales. Multiple scales can be analysed without interactions between them or a cross-scale analysis can be performed that accounts for interactions between scales^10^. Furthermore, adverse effects appearing outside the TOA case study area (off-site effects) may offset any gains stemming from a TOA-informed policy^11^. Second, recognition of social interactions and cultural values is needed to assure representation of beneficiaries and non-beneficiaries relevant to the topic at hand, that is, distributional justice^9,10^. Representation among stakeholders and their involvement in the design and implementation of a TOA can increase the legitimacy of its findings, assure that the data used are relevant to the context and thus enhance adoption of a study’s findings^12^. Third, validation and acknowledgement of uncertainty in both data and model estimates increase the robustness of a TOA and can facilitate risk-based decision-making^13–15^.

Previous literature reviews on TOA in agriculture adopted a ‘storytelling’ approach, where key studies were selected from the literature to discuss research trends. However, given the wide scope of TOAs applied in the context of agriculture, a systematic review could reveal the variety of approaches used and potential knowledge gaps, as well as the indicators that were studied and by which methods, ultimately facilitating the comparability of results.

Here we report on the TOA indicators, methodology and analysis used in 119 peer-reviewed articles. Descriptive statistics are used to characterize articles based on the extent to which they considered (1) indicators relevant to environmental and socio-economic services, (2) multiple spatial scales and their interactions, (3) the comprehensive involvement of stakeholders, and (4) the validity of trade-offs and recommendations in the context of associated uncertainties and risks (see Table 1 for further details). Finally, a cluster analysis shows which indicators were frequently studied together and which TOA methods were associated with each cluster.

**Table 1:** A generic description of the criteria that were logged during this systematic review

Criterion	Generic description
TOA method	The method (spatially explicit simulation (M1), simulation (M2), optimization (M3), cost–benefit analysis (M4), econometrics (M5), qualitative (M6), literature review (M7), GIS (M8) and other (M9)) applied in the case study
TOA scale	The spatial scale (field, farm, regional, national, multi-country and global) of the case study area for which the TOA is performed
Discipline	The spatial scale (the same spatial scales as for the TOA criterion) at which modelling was performed or data were collected in the six disciplines relevant to agricultural production and the provision of services (crop, livestock, economic, environment, fisheries and forestry)
TOA indicators	Indicators that were assessed in the TOA: 24 indicators were logged within the generic classes of agronomic (A: yield, yield stability, self-sufficiency, land use efficiency and input efficiency), economic (E: profitability, poverty, market supply or demand, labour productivity and assets), human health (H: nutrition, health, gender equity, food security and empowerment) and sustainable resource management (S: water quantity, water quality, soil nutrients, soil erosion, soil organic carbon, land use, greenhouse gases, energy and biodiversity)
Stakeholder type inclusion	Whether, and if so which, stakeholders were included in the case study (local beneficiaries and non-beneficiaries (Lb), experts (Ex), government (Gv), farmers (Fm), distant beneficiaries and non-beneficiaries (Db), academics (Ac), private sector (Pv) and environmental organizations (Eo))
Stakeholder implementation	How the stakeholders were included in the case study (validation (Vd), valuation (Va), co-development (Cd) and consultation (Co))
Scenario	Whether a scenario was considered in the case study and, if so, which type of scenario (climate (1), policy (2), behavioural (3), demographic (4), economic (5), resource use (6), other (7) and none (8))
System border	Whether biophysical or administrative boundaries were used to define the TOA case study area
Off-site	Whether off-site effects (those occurring outside the case study area) were considered in the TOA
Uncertainty	Whether uncertainty has been acknowledged and accounted for
Validation	Whether a validation was performed of the results obtained by data collection, the modelling procedure or the outcomes and recommendations from the TOA
Risk analysis	Whether the inference from the TOA and associated recommendations account for risk
Cross-scale analysis	Whether models address cross-scale TOA by summing or aggregation from lower to higher levels (aggregative) or by simultaneous quantification at multiple levels (interactive)

For further details, see Supplementary Table 1.

The aim of this study was thus to provide an overview of the peer-reviewed literature on TOA in the context of agriculture using a systematic approach. For this purpose, we sought to define how trade-offs in agriculture are conceptualized, characterized and analysed in the TOA literature. Based on these findings, we have identified common gaps in the implementation of TOA.

## Results

**Extended Data Fig. 1:**
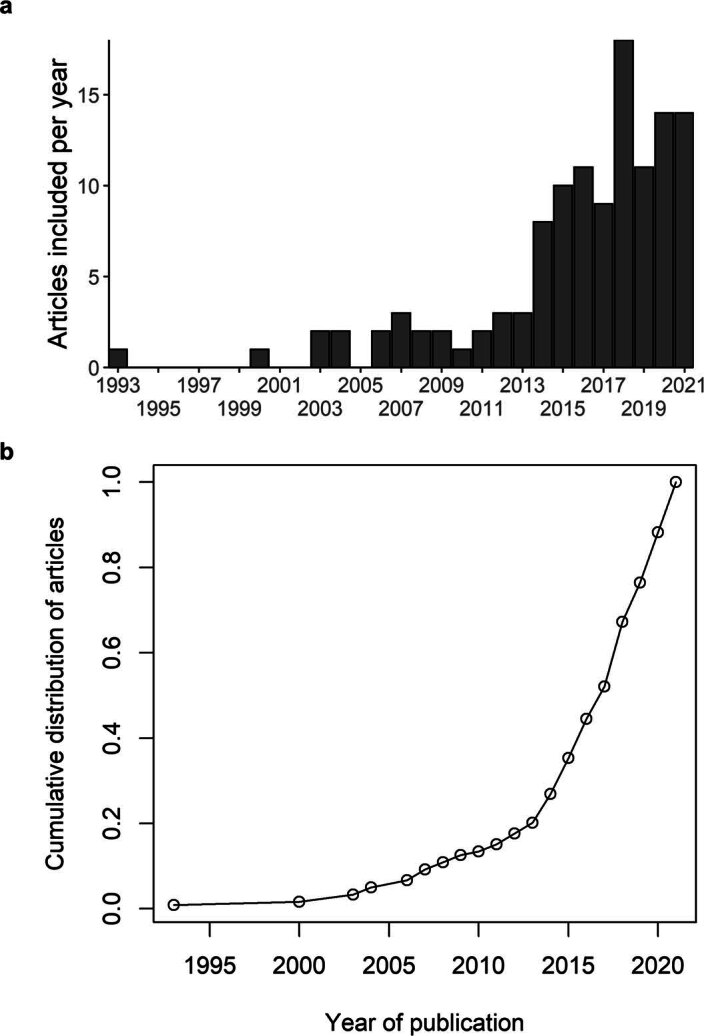
Articles per year of publication. Number of articles by publication year (**a**) and its cumulative distribution (**b**).

The distribution of publication dates for the articles in the sample was mainly centred in the years 2015–2021 (Extended Data Fig. 1a). Specifically, 73% of the articles were published after 2014, which indicates an increasing research effort directed towards TOAs in an agricultural context (Extended Data Fig. 1b).

### Common interrelationships and co-occurrences among TOA indicators

**Fig. 1:**
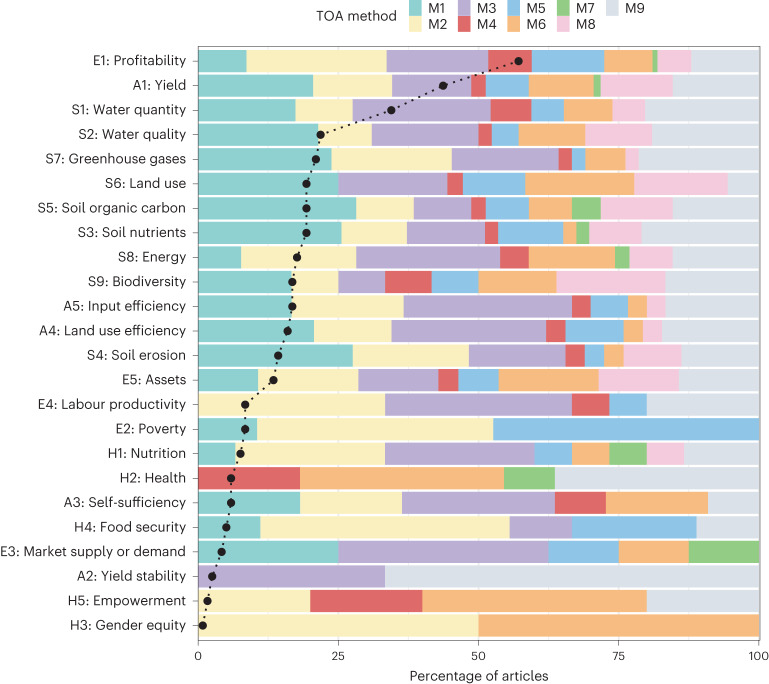
Articles that include TOA indicators. Percentage of articles that include a TOA indicator (black dotted line and circles) and the share of each TOA method M1–M9 used to study that indicator (coloured bars). The prefixes of the TOA indicators refer to their class association (A, E, H, S) and number of occurrence within that class as provided in Table 1. Table 1 also describes the TOA methods M1–M9.

**Extended Data Fig. 2:**
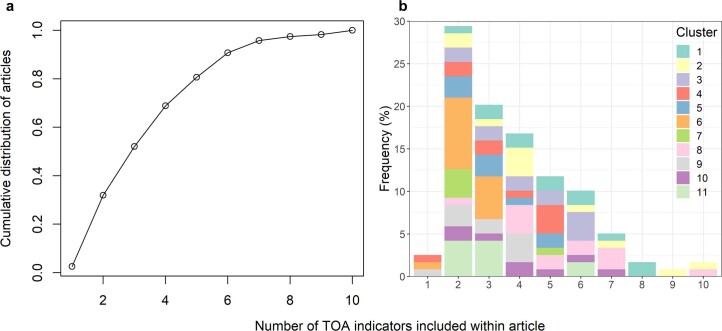
Figures on the number of trade-off analysis (TOA) indicators considered. Cumulative distribution of articles per number of TOA indicators included within an article (**a**). Frequency (%) of the number of TOA indicators included within an article, color-coded by cluster as specified in Fig. 2 in the main text (**b**).

The articles examined included a median of 3.8 ± 1.9 (s.d.) TOA indicators, ranging from 1 to 10. Based on the cumulative distribution, 52% of the articles included three or fewer TOA indicators, while 90% included six or fewer TOA indicators (Extended Data Fig. 2a). The most prevalent indicators across all articles were ‘profitability’ (57%, economic), ‘yield’ (44%, agronomic) and ‘water quantity’ (34%, sustainable resource management). The second most common set of indicators encompassed a selection of biophysical (for example, ‘water quality’ and ‘greenhouse gases’), agronomic (for example, ‘input efficiency’ and ‘land use efficiency’) and economic indicators (for example, ‘assets’), ranging between 13% and 21% (Fig. 1). The remaining TOA indicators were used less frequently and related to economic (that is, ‘labour productivity’ and ‘poverty’), human health (for example, ‘nutrition’, ‘health’ or ‘food security’) and agronomic (that is, ‘self-sufficiency’) aspects, representing a share of 5–6% (Fig. 1). Rarely considered TOA indicators (less than 5%) included ‘market supply or demand’ (economic), ‘yield stability’ (agronomic), ‘empowerment’ and ‘gender equity’ (both human health; Fig. 1).

**Fig. 2:**
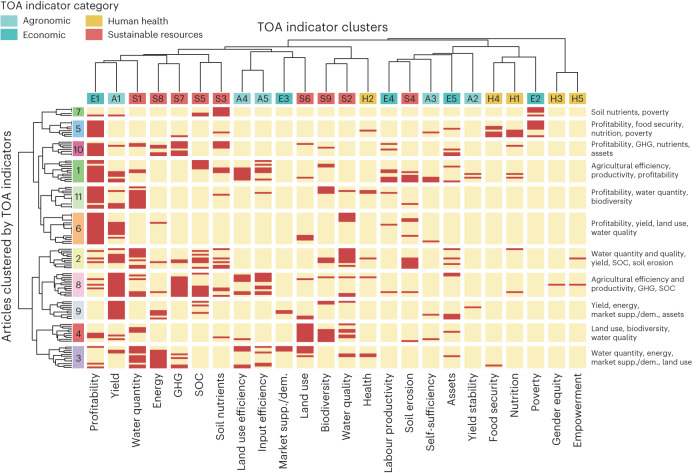
Clustering of articles. The articles were clustered by TOA indicator (row-wise) and TOA indicator clusters (column-wise). The associations of articles with clusters are indicated by the colours and labels on the left of the figure; the colours are arbitrary. TOA indicator clusters (top *x* axis) are specified by colour, corresponding to the main indicator categories (legend in top left of the figure), and their name (bottom *x* axis). The matrix indicates whether a TOA indicator has been included in an article (red) or not (beige). The labels on the right list the main TOA indicators included in each cluster. GHG, greenhouse gases; SOC, soil organic carbon; supp./dem., supply or demand.

The articles were grouped into 11 clusters, depending on which TOA indicators were assessed (left *y*-axis dendrogram in Fig. 2). These clusters show a dominant theme based on the co-occurrence of TOA indicators (right *y-*axis in Fig. 2). For example, in cluster 7, ‘poverty’ was studied in conjunction with ‘soil nutrients’, whereas in cluster 5, ‘poverty’ was studied in conjunction with ‘profitability’, ‘food security’ and ‘nutrition’. The clustering of articles by TOA indicator reveals which TOA indicators are often studied together. Indicators of ‘profitability’ and ‘yield’ were the most commonly used (Figs. 1 and 2) and were generally combined with case-specific environmental and social indicators (Fig. 2). This suggests that agronomic and economic viability are conditional for the exploration of improvements in agricultural system sustainability. The cluster with the largest number of articles (cluster 6, Fig. 2) concerned agricultural production and water quality. This highlights the strong focus on solving pressing issues related to pollution by surplus nutrients from fertilizers and manure.

The clustering of TOA indicators (top *x*-axis dendrogram in Fig. 2) shows that for 50% of the indicators, the indicator closest in the dendrogram belongs to the same category (sustainable resource management, agronomic, economic or human health). In particular, four out of five human health indicators were studied in isolation from other indicators, forming closely paired branches (top *x*-axis dendrogram, orange colour, in Fig. 2).

**Fig. 3:**
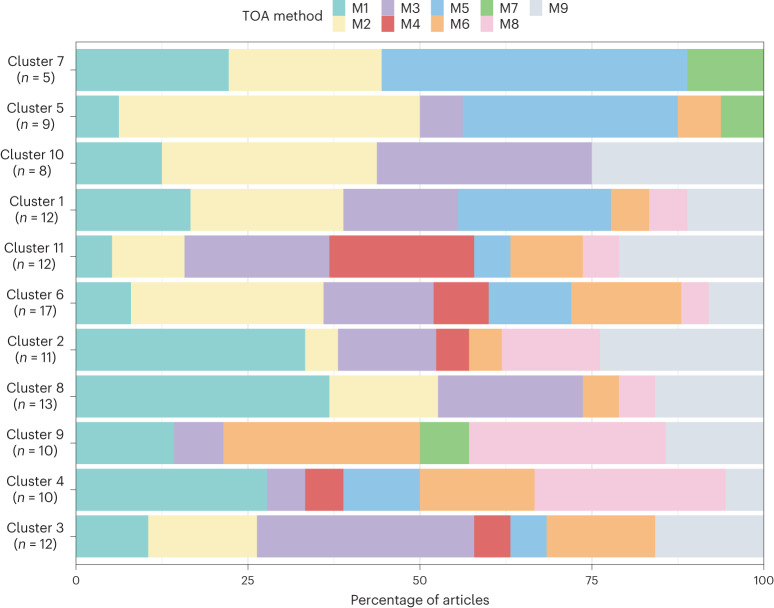
The share of each TOA method by cluster. Cluster associations are as per Fig. 2 and the number of articles within each cluster is given by *n*.

The application of TOA methods varied across different TOA indicators and clusters. For example, the TOA indicators ‘labour productivity’, ‘empowerment’, ‘gender equity’ and ‘yield stability’ lacked cases involving spatially explicit methods (M1 or M8; Fig. 1). This same observation applies to the clusters in which these TOA indicators belong (Fig. 3). While the absence of spatially explicit methods for social indicators such as ‘empowerment’ and ‘gender equity’ is expected, given that their spatial dimension is often disregarded, it is worth noting that gender and empowerment may relate to the spatial distribution of fields and resources in the landscape. For instance, their distance from the location of the homestead or decision-making processes regarding the (distribution of) use and ownership of these resources. Clusters of articles associated with ‘yield’, ‘energy’, ‘biodiversity’ and ‘land use’ exhibited a high use of GIS (M8), qualitative (M6) and other (M9) methods, with fewer articles applying optimization methods (M3; Fig. 3). Lastly, an interesting anomaly is the ‘health’ indicator, where methods M1–M3, encompassing (spatially explicit) simulations and optimization methods, were conspicuously absent (Fig. 1).

### Frequency of criteria levels

**Fig. 4:**
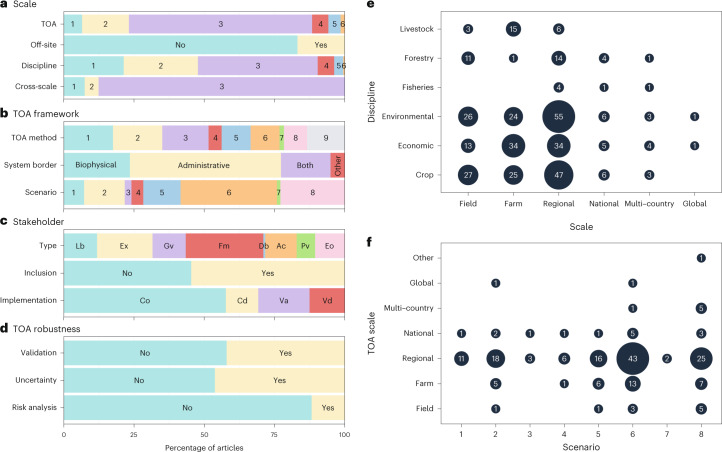
The relative frequency of each level within a criterion. **a**, Criteria related to the scale of the analysis. TOA: the spatial scale at which the TOA was conducted. The numbers refer to the spatial scales of field (1), farm (2), regional (3), national (4), multi-country (5) and global (6). Off-site: whether off-site effects have been considered in the TOA. Discipline: the spatial scale at which modelling or data collection was performed for a discipline. The numbers refer to the spatial scales detailed above for TOA. Cross-scale: whether aggregative (1), interactive (2) or no cross-scale modelling was performed (3). **b**, Criteria related to the TOA framework. TOA method: the methods used to perform the TOAs. The numbers refer to the TOA methods M1–M9 defined in Table 1. System border: which boundaries were used to define the TOA case study area. Scenario: whether the article considered a scenario and, if so, which type of scenario. The numbers refer to the scenarios 1–8 defined in Table 1. **c**, Criteria related to stakeholders. Type: whether local beneficiaries and non-beneficiaries, experts, government, farmers, distant beneficiaries and non-beneficiaries, academics, private organizations or environmental organizations were involved. Inclusion: whether the study included stakeholders. Implementation: whether stakeholders were involved in consultation, co-development, valuation or validation. **d**, Criteria related to TOA robustness: whether the article performed a validation, risk analysis or acknowledged uncertainty. **e**, The frequency (shown in the circles) for each spatial scale at which the modelling or data collection was performed for a given discipline. **f**, The frequency (shown in the circles) at which an article considered a given scenario in TOA for each spatial scale. The scenario numbers 1–8 are defined in Table 1.

The majority of TOAs were conducted at regional (65%) and farm (17%) scales, followed by field (7%) and national (6%) scales. The TOAs conducted at multi-country (4%) and global scales, along with ‘other’, accounted for only a small proportion of the analyses (Fig. 4a). The spatial scales for TOAs differed from the scales at which modelling was performed or data were collected, with the farm and field scale contributing to a combined share of 48%. Of the articles considered, 12% implemented cross-scale analyses and 17% considered off-site effects (Fig. 4a). Case study areas were predominantly delineated using administrative borders (54%), followed by biophysical delineation (24%), with 18% of the articles using both methods (Fig. 4b).

Including a scenario in the TOA allows investigation of the effect of a postulated event or driver on the TOA indicators. In our analysis, scenarios focusing on climate, behavioural or demographic change accounted for 14% of the articles, while scenarios involving alternative intensities of resource use constituted 37% of the articles. Scenarios were absent in 25% of the articles (Fig. 4b). Over half of the articles included stakeholders in their analysis, with a relatively equal spread across stakeholder types, except for ‘distant beneficiaries and non-beneficiaries’, which were under-represented. Farmers and experts constituted a larger share (48%) compared to other categories (Fig. 4c). Stakeholders were mainly involved in consultation and valuation, with co-development and validation implemented in less than 25% of the articles considered (Fig. 4c). Overall, the robustness of the TOA results was not widely considered, as the criteria ‘uncertainty’ and ‘validation’ were logged for less than 50% of the articles. Articles incorporating risk analysis constituted 12% of the sample (Fig. 4d).

### Links between spatial scales and criteria

Of the articles considered, ‘livestock’, ‘fisheries’ and ‘forestry’ accounted for a relatively small share (16%) compared with ‘crop’, ‘economic’ and ‘environmental’ disciplines. For the livestock discipline, modelling and data collection were predominantly carried out at the farm scale, while for forestry, they were primarily conducted at the field or regional scale (Fig. 4e). For the economic discipline, modelling and data collection were evenly distributed between the farm (*n* = 34) and regional (*n* = 34) scales (Fig. 4e), in contrast to the overall share of these scales across all of the articles, where ‘regional’ constituted 65% and ‘farm’ constituted 17% of the articles (Fig. 4a). In general, for a large share of the reviewed articles, data were collected and modelling was performed at the field and farm scales, but the TOA was conducted at the regional scale. These findings show that, before the TOA, some form of aggregation occurs in the majority of the reviewed articles. Regarding the spatial scale at which the TOA was conducted for articles including a scenario, two observations can be made. First, all of the scenarios (except the resource use scenario) were rarely studied at scales larger than the national scale. Second, the climate, behavioural and demographic change scenarios were almost exclusively studied at the regional scale (Fig. 4f). These results show that few studies investigated how scenarios unfolding at smaller or larger scales affect the indicators at the TOA scale.

### Multi-scale, cross-scale and robustness criteria

**Fig. 5:**
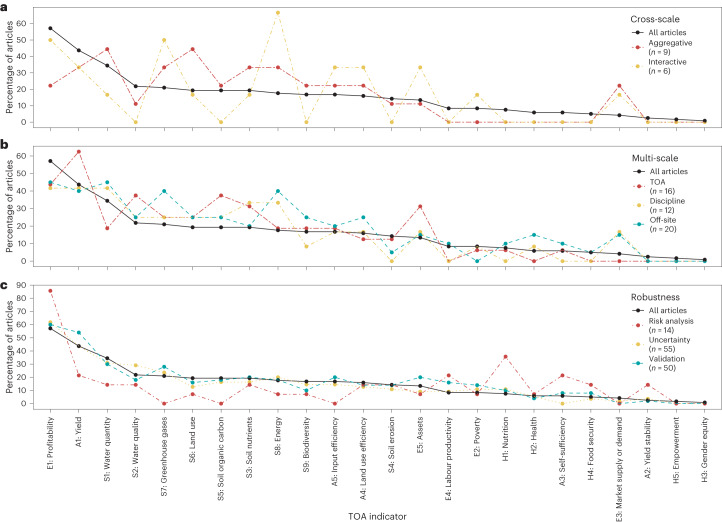
Percentage of articles that include a TOA indicator. The percentages of all reviewed articles and subsets of articles that include specific TOA indicators. **a**–**c**, The subsets comprise articles that included cross-scale (**a**), multi-scale (**b**) and robustness (**c**) criteria. In **b**, TOA refers to articles in which the TOA was conducted on multiple spatial scales, ‘Discipline’ refers to articles that considered multiple spatial scales for modelling or data collection, and ‘Off-site’ refers to articles in which effects outside the TOA case study area were considered.

Figure 5 shows the percentage of articles that include a TOA indicator (black line, the same as shown in Fig. 1). The articles were then divided into subsets according to whether they included a cross-scale, multi-scale or robustness criterion. The coloured lines represent the percentage of articles in the subset that include a specific TOA indicator. With the exception of indicators rarely included in all articles (for example, those related to nutrition or health), most TOA indicators were present in articles adopting a cross-scale modelling framework (Fig. 5a). These findings occur despite the overall low number of articles (<20%) reporting cross-scale analyses (Fig. 5a). Notably, articles applying an interactive modelling framework did not include ‘water quality’, ‘soil erosion’, ‘soil organic carbon’ and ‘biodiversity’, despite these indicators having a relatively high frequency across all articles (Fig. 5a).

Across all articles, 17% considered off-site effects (Fig. 4a). Notably, the ‘poverty’ and ‘soil erosion’ indicators were under-represented in articles considering off-site effects (Fig. 5b). Eight indicators were excluded in articles considering multiple spatial scales in modelling or data collection (‘discipline’ in Fig. 5b). This finding is particularly striking for ‘biodiversity’, given that it constitutes a large share of spatially explicit TOA methods (Fig. 1).

Thirteen per cent of articles reported TOA on multiple spatial scales, with seven indicators excluded in these cases (‘TOA’ in Fig. 5b). Among the excluded indicators, those related to human health dominated (except for ‘nutrition’). For certain indicators, these findings are to be expected. For instance, market supply or demand (economic) is irrelevant at low geographical scales (field and farm) as prices are determined at the regional (local), national or international scale. The articles that included a risk analysis showed stark contrasts between TOA indicators with respect to their representation relative to all articles. Economic and human health indicators were particularly over-represented, while ‘yield’, ‘input efficiency’ and a set of biophysical indicators were under-represented (Fig. 5c). For articles in which uncertainty was acknowledged or validation was performed, no indicators were over- or under-represented relative to their inclusion across all articles (Fig. 5c).

## Discussion

### Limitations on the inclusion of TOA indicators

Recent reviews on TOA have stated that there is little to no representation of indicators related to social interactions, justice and gender issues in TOAs for agricultural systems^5,6^. These studies referred in particular to intra-household equity, asset ownership, health, education and nutrition. Our results also demonstrate that social and cultural TOA indicators are largely absent, mostly considered in isolation and studied by statistical approaches. These findings are probably a result of the limited data availability and the inability of TOA methods to include socio-cultural indicators for features and processes that are difficult to capture quantitatively^16,17^. We further note a similarly low frequency for the following indicators: food security, self-sufficiency and yield stability. These findings raise questions about the rationale behind the selection of TOA indicators. That is, the prevalent use of profitability and crop yield as primary indicators reflects the focus on profit and crop yield maximization in the literature^5^. The outcomes and priorities of a TOA depend on the chosen objectives and indicators. Alternative indicators might therefore facilitate a more comprehensive analysis of the delivery of environmental, economic and socio-cultural services from agriculture. One illustrative example is the metric ‘nutritional yield’, defined as “the number of hectares required to provide sufficient quantity to fulfil 100% of dietary reference intake for a nutrient for one adult”^2^. Nutritional yield thus allows the assessment of land use efficiency in both agronomic and social terms. Integrating nutritional yield into TOA in the context of subsistence agriculture could unveil the need for changes in farmers’ crop plans to balance food security and economic profitability objectives.

### TOA methodologies

The formulation of research objectives, questions and methodology determines the information base that a TOA can provide^16,18^. Decisions regarding TOA objectives and methodology determine the degree to which scales, disciplines and indicators are compartmentalized. In addition, these decisions influence the range of interventions and scenarios explored for alternative agro-environmental management of land, resources and technologies^7,18^. The results of our analysis reveal associations between TOA methods and indicators, indicating common gaps, such as the absence of articles reporting the use of spatially explicit methods to study the indicators ‘human health’ and ‘yield stability’. Studying these indicators in a spatially explicit manner could allow for targeted land use planning at the local scale. For instance, Prestele and Verburg demonstrated that spatially explicit analysis of climate-smart agriculture adoptions unveils local-scale trade-offs affecting yield and soil carbon sequestration at an aggregated scale^19^. Our results also underscore expected patterns, with socio-economic indicators predominantly studied through statistical approaches and qualitative methods. These methods, static and based on existing datasets, differ from mechanistic models, which allow extrapolation and ex ante assessment under alternative future scenarios. Simulations based on mechanistic models hold the potential to explore scenarios that minimize trade-offs between indicators^3,7^. However, the validity of this kind of optimization depends on having sufficient understanding of relevant processes and feedbacks in the socio-environmental system^3^. For example, while crop models vary in their capacity to assess climate change impacts, they share common limitations, such as inadequate representation of low-intensity agricultural systems^20^. We found that a description of study limitations in the context of the TOA framework, for example, excluded aspects, was often absent. Ideally, models and associated uncertainties would be assessed in the design phase of the TOA. This could ensure the availability of adequate information for quantifying all desired parameters at the desired resolution, allowing the study to comprehensively represent the agricultural system. Such an approach is crucial to guide planning in future management decisions aligned with research objectives^17^.

### Involvement of stakeholders and practical application of TOA results

One recurring concern in the literature is the frequent omission of stakeholders at the onset of the TOA, potentially limiting the practical application of TOA results^6,8^. Our findings partially support these concerns, given that co-development with stakeholders was observed in only 10% of the articles. However, making a definitive statement on equal representation among stakeholders proved challenging as there was generally an absence of a systematic inventory outlining the relevance of different stakeholders to the decision-making process based on their interests and influence^21^. Our analysis shows that farmers and experts were the primary stakeholders included in the articles. Nonetheless, the omission of distant beneficiaries and non-beneficiaries is noteworthy as they are likely to be relevant to the decision-making process in numerous cases, especially when off-site effects are considered in TOAs conducted on multiple scales.

### Multi- and cross-scale analysis

Depending on the research objectives, the TOA literature underscores the importance of acknowledging processes across scales and including them in research^3,6–9,22^. In many of the articles, data were collected or modelling was performed at field and farm scales, yet the TOA was conducted at the regional scale. This highlights an opportunity for multi-scale TOA analysis, potentially enhancing the relevance of TOA studies to policy. For example, bilevel optimization is a promising approach to facilitating nested decision-making processes at different scales. In this approach, the solution at the higher level (for example, larger spatial scale) depends on the solution at the lower level (for example, smaller spatial scale). Bostian et al. demonstrated the application of this methodology in recognizing multiple spatial scales inherent to non-point pollution regulation^23^. However, the restricted application of cross-scale analysis in our sample (12%) shows the limited extent to which TOA in agriculture captures the hierarchical nature of social, cultural, environmental, economic and agronomic processes.

Furthermore, 17% of the articles considered effects outside the TOA case study area, considering off-site effects in a diverse array of subjects, including transnational emission permits, water trading and increased demand for scarce resources, anticipated to influence their shadow prices^24–26^. However, off-site effects might have feedbacks, such as dependencies between alternative production systems within a supply chain^27^. In such cases, the delineation of the system boundary must be considered in the context of these feedbacks to ensure their inclusion within the system. In cases where off-site effects do not have feedbacks, these can be classified as ‘teleconnections’, denoting processes whose cause and effect are widely separated^28^. A case in point is a study of the water quality of the Danube River, in which distant beneficiaries and non-beneficiaries, represented by an international committee, were considered in the TOA^29^. The results also show that climate, behavioural and demographic scenarios were rarely assessed at lower or higher scales (compared to the regional scale). This underscores that the extent to which these scales are relevant to TOA is understudied and merits further research. For example, generic methods, such as the carbon^30^ or water^31^ footprint, can provide a broad assessment of which off-site effects at larger scales are relevant to TOA outcomes. These approaches may facilitate the inclusion of underlying causes, the involvement of more inclusive stakeholders and account for leakage effects, such as the expansion of agricultural lands beyond the TOA case study area^32^.

Ideally, a TOA methodological framework is conceptualized such that (1) it recognizes multi- and cross-scale interactions where applicable, (2) the system boundary aligns with substantiated biophysical and relevant socio-institutional boundaries, and (3) it recognizes the heterogeneity in which scales and associated consequences are perceived as well as valued by different stakeholders^10^.

### Robustness of TOA results

The risk associated with TOA extends across spatial, temporal and jurisdictional scales, carrying implications for the dissemination of TOA results^13^. The under-representation of ‘yield’ in articles considering risk analysis highlights the dichotomy between yield and profitability as the most prominent indicators. That is, risk analysis appears to be mainly associated with the economic domain^5^. However, it is important to recognize that the evaluation of risk and the formulation of relevant strategies (risk aversion, mitigation or offsetting) are critical for farmers adopting system transformations, such as alternative forms of land use to mitigate inputs and associated greenhouse gas emissions. Integrating risk into TOA enables the study of the policies and incentives necessary for achieving whole-system transformations towards sustainable agricultural practices^13,14^. Decision-making under uncertainty becomes interpretable when recommendations are accompanied by an assessment of associated risks. Ideally, these risks are context-specific. For example, Hochman et al. provided TOA results on crop rotations alongside a minimum risk threshold quantified as the highest gross margin for the poorest 20% of years^33^.

While a moderate number of the articles considered uncertainty, only a few articles quantified changes in trade-offs as a function of uncertainty. The inclusion of stochastic components and the associated uncertainty inherent in biological systems could facilitate a more realistic description of outcomes, proving valuable for decision-making^13,15^. Varying input data or model parameterization within an expected range could reveal the sensitivity of results. For instance, when climate scenarios are used, realizations of these scenarios can be used to assess the stochasticity of the objectives for which the TOA is implemented^34^. This approach enables the acknowledgement of both the frequency and pattern of stochastic events, including extreme weather events, and their impact on TOA outcomes. Consequently, an analysis of the adaptability of a farming system would not solely rely on optimal solutions given the mean output but would also account for associated variability and unexpected events^15^. However, it is crucial to contextualize the effect of stochasticity. For example, the relative impact of model or parameter uncertainty on optimization outcomes has been shown to vary depending on the prioritization of objectives and site conditions^35^.

### Limitations of this study

An important limitation of our review lies in the use of ‘trade-off analysis’ as a single term in our Web of Science search string. There are research areas that address trade-offs and synergies across various disciplines, scales and methods without explicitly using the term ‘trade-off analysis’ to describe their research objectives. Examples include the ‘food–energy–water nexus’ literature^36^, as well as research under the auspices of the Agricultural Model Intercomparison and Improvement Project (AgMIP) (https://agmip.org/) and the Food, Agriculture, Biodiversity, Land-Use and Energy (FABLE) Consortium^37^. Both AgMIP and FABLE are particularly concerned with the relevance of TOA to policy. AgMIP explicitly states the use of “multiple scenarios and models to assess and probabilistically manage risk”^38^. Given the focus of these studies on global and regional assessments, we anticipate that our findings for those spatial scales could be affected. Indeed, the identified gaps in TOA implementation need to be viewed in the context of our sample, which mostly comprises studies in which modelling or data analysis was performed up to the regional level and TOA at the regional scale.

The method used to log the occurrence of pre-set criteria not only affects the variance within a criterion but also influences its abundance. For example, Sanon et al. included a large number of TOA indicators that were all classified under ‘biodiversity’^29^. Thus, binary criteria logging does not capture the intensity with which a criterion is considered, a well-known phenomenon in the field of ecology^39^. This limitation may have resulted in the underestimation of both the intensity with which certain TOA indicators and their classes have been studied (Fig. 1) and the total number of TOA indicators considered per article (Extended Data Fig. 2).

## Conclusions

Based on our analysis, it is possible to identify some actions that would increase the contribution of TOAs to SDG-aligned agricultural landscapes.

For instance, future studies should include multi- and cross-scale effects when relevant to the research objectives. We have identified an opportunity for multi-scale analysis, given that many studies aggregated farm- or field-scale data before performing TOA at a regional scale. As the inclusion of multiple scales, indicators and methods may in some cases reduce the generalizability of results and make them more context-specific, an alternative would be to discuss the anticipated implications of multi- and cross-scale effects on the study findings.

Furthermore, the relevance of TOA to society and policy can be improved by formulating research objectives such that TOA indicators lie within the scope of frameworks such as the SDGs. The most frequent indicators were biophysical or informed by profit maximization theory (for example, profitability and yield). However, indicators relevant to human well-being, security and farm resilience (for example, empowerment, nutrition and yield stability) occurred less frequently. To aid the interpretation of TOA results, the rationale behind the TOA methodology that is used to assess indicators should be listed together with a critical review of how the agricultural system under study is represented and what is excluded as a consequence.

In the reviewed articles, the most consulted stakeholders were farmers and experts, stakeholder co-development and validation were rare, and scenarios were predominantly based on resource use with little consideration of off-site effects. These findings suggest that TOAs mostly explore alternative management across a set of farms rather than policies and incentives that would facilitate whole landscape and food system transformations.

Agricultural policy- and decision-making carry an inherent risk. TOAs will become more operational when they evaluate associated risks and list strategies to manage these risks. This process could promote the robustness of quantified trade-offs with respect to the associated uncertainty of data and variability in outcomes. Finally, an inventory of stakeholders that are relevant to the decision-making process and their respective roles in the study would provide legitimacy of results. While this element has already been recognized in the literature^12,29^, some of the shortcomings that we have identified here would probably occur less frequently, particularly the lack of stakeholder inclusion and the over-representation of specific stakeholder types and methods of stakeholder engagement.

Closer adherence to these guidelines could enhance the relevance of TOA to the scientific community, policy-makers and farmers.

## Methods

We followed the approach of Lautenbach et al. and Seppelt et al. in their systematic review of the literature on ecosystem services^9,22^. The generic structure involved (1) the identification, screening and selection of relevant peer-reviewed literature from a global repository, (2) formulation of the criteria against which to evaluate each article (Table 1 and Supplementary Table 1), and (3) descriptive statistics and cluster analysis to assess common interrelationships between criteria and identify knowledge gaps.

We used the following search string “ALL=agricultur* AND (“trade off* analysis” OR “trade-off* analysis” OR “tradeoff* analysis”)” in the Web of Science (on 14 September 2021) to identify peer-reviewed articles in English reporting TOA. We found 153 articles with publication dates spanning from 1993 to 2021. We excluded studies that mentioned the existence of trade-offs but did not assess relationships between indicators. For this reason, review and opinion papers were considered off-topic and were excluded from the search results. Furthermore, methodological papers that did not involve a case study were also excluded, leading to a total sample of 119 articles.

We selected criteria based on current TOA research^5–9,16,22^ and recorded information on these criteria that were relevant to the conceptualization, characterization and analysis of trade-offs in agriculture (research objective 1). Briefly, the criteria included the type of TOA methods used, the spatial scales at which the analyses were performed and/or data collected, the indicators assessed in the TOA, which stakeholder types were included as well as how the stakeholders were engaged in the case study, whether the case study included alternative scenarios and of what type, how the case study area was delineated, whether effects outside the case study area were considered, and whether the case study acknowledged and accounted for uncertainty, validated results or performed a risk analysis. To assess whether cross-scale analyses were performed in case studies, we adopted the definition of Kanter et al., who distinguished between model frameworks that aggregate outputs at lower scales to use as inputs at higher scales (aggregative) and model frameworks that have submodels operating at different spatial and temporal resolutions (interactive)^6^. Thus, whereas an aggregative model framework follows a sequential approach, an interactive model framework performs analysis across scales simultaneously, allowing for interactions between scales and emergent indicators at higher levels. Furthermore, descriptive information was collected for three criteria: the agricultural system(s) studied, agricultural activities and knowledge gaps reported in the discussion section of the article. All of the criteria are listed in Table 1 with a generic description. We refer the reader to Supplementary Table 1 for more detailed information on the criteria. Based on these criteria, knowledge gaps were then assessed through descriptive statistics and cluster analysis (research objective 2).

The decision of which TOA indicators to include is a major methodological decision in TOA as it determines which interrelations are considered and analysed, and therefore which trade-offs and synergies can be identified. We anticipated thematic clusters of TOA indicators based on the discipline, scale, geography and method considered. To identify co-occurrences between TOA indicators, we performed hierarchical Ward clustering to group articles by TOA indicators as well as the TOA indicators themselves based on the Jaccard similarity coefficient^40^. Through the use of the Jaccard similarity metric, we accounted for the double-zero problem. Namely, the absence of a TOA indicator in two articles does not indicate a similarity, whereas its presence does^9^. For the clustering of articles by the TOA indicators used, the number of clusters to be retained was decided by the ‘elbow’ method based on the Mantel correlation between the data for each cluster and the raw distance matrix^40^. For the clustering of TOA indicators, the dendrogram was not cut to visualize common co-occurrences for all of the TOA indicators.

Criteria were logged in a Microsoft Office Excel (2021) spreadsheet (Supplementary Data 1). The data collected during this systematic review were further analysed and visualized in R (ref. ^41^). Data handling, visualizations and analysis were performed using the following R packages: tidyverse^42^, dendextend^43^, cluster^44^, vegan^45^ and pheatmap^46^.

### Reporting summary

Further information on research design is available in the Nature Portfolio Reporting Summary linked to this article.

### Supplementary information

Supplementary Information Supplementary Table 1, Figs. 1–9 and a list of articles included in the systematic review.

Reporting Summary

Supplementary Data 1 Criteria assessed in the systematic review. This file was used to perform the analysis and create the figures.

## Data Availability

The dataset created has been made available as extended data.

## Code availability

The code created for data handling, analysis and visualizations is available on request.
