# Supplementary material for: A systematic review of the methodology of trade-off analysis in agriculture
Source: Nat Food. 2024 Mar 5;5(3):211–20. doi: 10.1038/s43016-024-00926-x (PMC10963264; doi:10.1038/s43016-024-00926-x)
Supplement: Supplementary file 1 — Supplementary Table 1, Figs. 1–9 and a list of articles included in the systematic review. [file 43016_2024_926_MOESM1_ESM.pdf]

nature food

Article

https://doi.org/10.1038/s43016-024-00926-x

A systematic review of the methodology of trade-off analysis in agriculture

In the format provided by the authors and unedited

Supplementary material

A systematic review on the methodology of trade-off analyses in agriculture

Breure, T.S.[1], Estrada-Carmona, N.[2], Petsakos, A.[3], Gotor, E.[3], Jansen, B.[4], Groot, J.C.J.[1*]

[1] *Farming Systems Ecology, Wageningen University & Research, Wageningen, Netherlands*
[2] *Bioversity International, Montpellier, France*
[3] *Bioversity International, Rome, Italy*
[4] *Institute for Biodiversity and Ecosystem Dynamics, University of Amsterdam, Amsterdam, Netherlands*
[*] corresponding author: jeroen.groot@wur.nl

Contents

1 Supplementary tables 2

2 Additional figures not referenced within manuscript 8
2.1 Articles by human development index 8
2.2 Geographical trends of articles on TOA in agriculture 11
2.3 Cluster analysis 14
2.4 Wordclouds 16

3 Publications used in the systematic review 20

4 References of the supplementary material 30

1 Supplementary tables

Table S1 – Definitions of the criteria logged during the systematic review and their associated levels

Criterion	Description	Levels
TOA method	Primary method(s) applied to generate values for the TOA indicators.	1=Simulation modelling (spatially explicit); 2=Simulation modelling (parsimonious); 3=Optimization methods; 4= Cost-benefit analysis/Economic surplus; 5=Econometrics; 6=Qualitative approaches; 7=Meta-analysis, systematic-review; 8=Spatial analysis, GIS, Remote sensing; 9=Other
TOA spatial scale	The spatial scale at which the trade-off indicators are evaluated	1= Field; 2=Farm; 3=Region; 4=National; 5=Multi-country; 6=Global; 7=Other
Discipline	At which spatial scale a discipline (Crop, Economic, Livestock, Environment, Fisheries, Forestry) is considered within the modelling/data collection/analysis.	1= Field; 2=Farm; 3=Region; 4=National; 5=Multi-country; 6=Global; 7=Other
	TOA indicators: Economic	
Profitability	Indicator that quantifies the financial gain (e.g. net revenue)	0=No; 1=Yes

Continued from previous page

Criterion	Description	Levels
Poverty	Indicator that quantifies the number of individuals below a set threshold at which they are not able to afford an adequate standard of living (e.g. number of farmers within the case-study domain that earn an income below a monetary threshold)	0=No; 1=Yes
Market supply demand	Indicator that quantifies the change in market supply or demand of a commodity	0=No; 1=Yes
Assets	Indicator that quantifies anything tangible/intangible that is owned/controlled by an individual/community to produce value and that is held to have positive economic value (e.g. cattle herd density within a farm)	0=No; 1=Yes
Labor productivity	Indicator that quantifies the production of a desired output as a function of the amount of labor (e.g. share of farmland to produce a specified number of calories within a region)	0=No; 1=Yes
TOA indicators: Sustainable resource management		
Biodiversity	Indicator that quantifies the variability among living organisms from all sources and the ecological complexes of which they are part (e.g. richness/evenness indices)	0=No; 1=Yes
Soil organic carbon	Indicator that quantifies the soil organic carbon stock within a given model domain	0=No; 1=Yes
Soil nutrients	Indicator that quantifies the stock of soil nutrients within a given model domain	0=No; 1=Yes
Soil erosion	Indicator that quantifies the removal/retention of soil surface material within a given model domain	0=No; 1=Yes
Water quality	Indicator that quantifies physical, chemical and biological characteristics of water	0=No; 1=Yes

Continued from previous page

Criterion	Description	Levels
Water quantity	Indicator that quantifies the volume of water within a given model domain (e.g. share of hydrological flow contributed to agriculture)	0=No; 1=Yes
Energy	Indicator that quantifies a variable relevant to energy within the model domain (e.g. usage/availability)	0=No; 1=Yes
GHG	Indicator that quantifies the discharge of greenhouse gases, such as carbon dioxide, methane, nitrous oxide and various halogenated hydrocarbons, into the atmosphere	0=No; 1=Yes
Land use	Indicator that quantifies arrangements, activities and inputs people undertake in a certain land cover type to maintain it or produce change	0=No; 1=Yes
TOA indicators: Human well-being		
Gender equity	Indicator that quantifies the fairness of treatment for women and men according to their respective needs	0=No; 1=Yes
Empowerment	Indicator that quantifies the enhancement of the assets and capabilities of diverse individuals and groups to function, engage, influence, and/or hold accountable the institutions that affect them.	0=No; 1=Yes
Food security	Indicator that assesses the reliable physical and economic access to nutritious and sufficient food	0=No; 1=Yes
Health	Indicator that quantifies the state of functioning optimally without evidence of disease	0=No; 1=Yes
Nutrition	Indicator that quantifies a (set of) variable(s) relevant to the food intake required for health and growth (e.g. calories to proteins ratio produced at a farm)	0=No; 1=Yes
TOA indicators: Agronomic		

Continued from previous page		
**Criterion**	**Description**	**Levels**
Yield	Indicator that quantifies the total harvest/production from a farm	0=No; 1=Yes
Yield stability	Indicator that quantifies the change in agricultural yield given a constraint or scenario	0=No; 1=Yes
Input efficiency	Indicator that quantifies the input/output ratio of variables relevant to production	0=No; 1=Yes
Land use efficiency	Indicator that quantifies the input/output ratio of a (set of) variable(s) relevant to arrangements, activities and inputs people undertake in a certain land cover type to maintain it or produce change for a spatial unit	0=No; 1=Yes
Self sufficiency	Indicator that quantifies the extent to which the given model domain unit (e.g. farm, region) can supply in its dietary/nutritional needs	0=No; 1=Yes
Stakeholders		
Stakeholder	Whether individuals, groups, organizations or sectors in society that have a clearly identifiable interest within the TOA context have been reported to be included within the case-study	0=No; 1=Yes
Experts	–	0=No; 1=Yes
Governments	–	0=No; 1=Yes
Farmers	–	0=No; 1=Yes
Academia	–	0=No; 1=Yes
Private sector	–	0=No; 1=Yes
Environmental organizations	–	0=No; 1=Yes

Continued from previous page

**Criterion**	**Description**	**Levels**
Local beneficiaries	Persons within TOA case-study area that do not fall under other categories (e.g. civil society stakeholders)	0=No; 1=Yes
Distant beneficiaries	Persons outside TOA case-study area (but are affected by TOA) that do not fall under other categories	0=No; 1=Yes
Stakeholder involvement	Which type of stakeholder engagement has been employed within the TOA	1=Consultation; 2=Co-development; 3=Valuation; 4=Validation
	Other	
Scenario	Whether a postulated sequence of events is used within the analysis and if so, which type of events are included	1=Climate; 2=Policy; 3=Behavioral; 4=Demography; 5=Economic; 6=Resource usage; 7=Other; 8=None
System border	Whether the TOA case-study area has been defined based on biophysical or administrative boundaries	1=Biophysical; 2=Administrative; 3=Both; 4=Other
Off-site	Whether off-site effects (those occurring outside the case-study area) have been considered within the TOA. E.g. environmental processes at larger scales/trade-mediated effects.	0=No; 1=Yes
Uncertainty	Whether a state of incomplete knowledge has been recognized, either due to a lack of information or a disagreement about what is known/knowable	0=No; 1=Yes
Validation	Whether the case-study performs a validation of results obtained by data collection, the modelling procedure or the outcomes and recommendations from the TOA	0=No; 1=Yes

Continued from previous page		
**Criterion**	**Description**	**Levels**
Risk analysis	Whether the inference from the TOA and any subsequent recommendations account for associated risks	0=No; 1=Yes
Cross-scale analysis	Whether models that inform the TOA have been integrated across scales; Aggregative=Model frameworks that aggregate model outputs at lower scales to use as inputs at higher scales; Interactive=Model frameworks that have sub-models operating at different spatial and temporal resolutions	1=Aggregative; 2=Interactive; 3=None
Data visualization	Visualization method applied to compare across TOA indicators	1=Tabular matrix; 2=Bar charts; 3=Scatterplots/Pareto-frontier; 4=Spider/radial/petal/polar diagrams; 5=Maps; 6=Line graph; 7=Other
Data type informing indicators	The type of data that has been used to inform the TOA analysis and therefore modelling of the relative changes of indicators	1=Primary; 2=Secondary; 3=Elicitation; 4=Proxy; 5=Models
ESS	Whether the study considers the TOA indicators within an ecosystem services methodological framework	0=No; 1=Yes
Farming system	Descriptive information on farming system	Aposteriori classification
Farm management	Descriptive information on management on farms considered within the TOA	Aposteriori classification
Knowledge gap	Descriptive information on remaining knowledge gaps based on TOA	Aposteriori classification
Synergy	Whether synergies are also identified within the TOA	0=No; 1=Yes

2 Additional figures not referenced within manuscript

2.1 Articles by human development index

With regards to the logging of criteria relevant to countries: Some corresponding authors listed affiliated institutes within multiple countries. In that case, the insitute listed as first affiliation and its respective location was registered. Furthermore, some articles considered a case-study that spanned multiple countries. In that case, all countries have been included. Global studies (n=1) have been excluded from all maps.

"The human development index (HDI) is composite index measuring average achievement in three basic dimensions of human development-a long and healthy life, knowledge and a decent standard of living" (UNDP, 2020). The HDI values for the year 2019 have been used, the classifications (low, medium, high, very high) are provided by the UNDP for each year.

Figure 3 shows that based on the countries that occure with a frequency > 1, the lead author's affiliation does not include countries in the lower two categories of the human development index (HDI) except for Kenya, Pakistan and Ethiopia (left-panel). However, countries where the case-study areas are located show a much larger share of countires in the lower two categories of the HDI (right-panel) as it also includes Zimbabwe, Senegal, Mali, Zambia, Tanzania, Ghana, Uganda, Rwanda, Mauritania, Guinea, Cameroon, Benin and Angola.

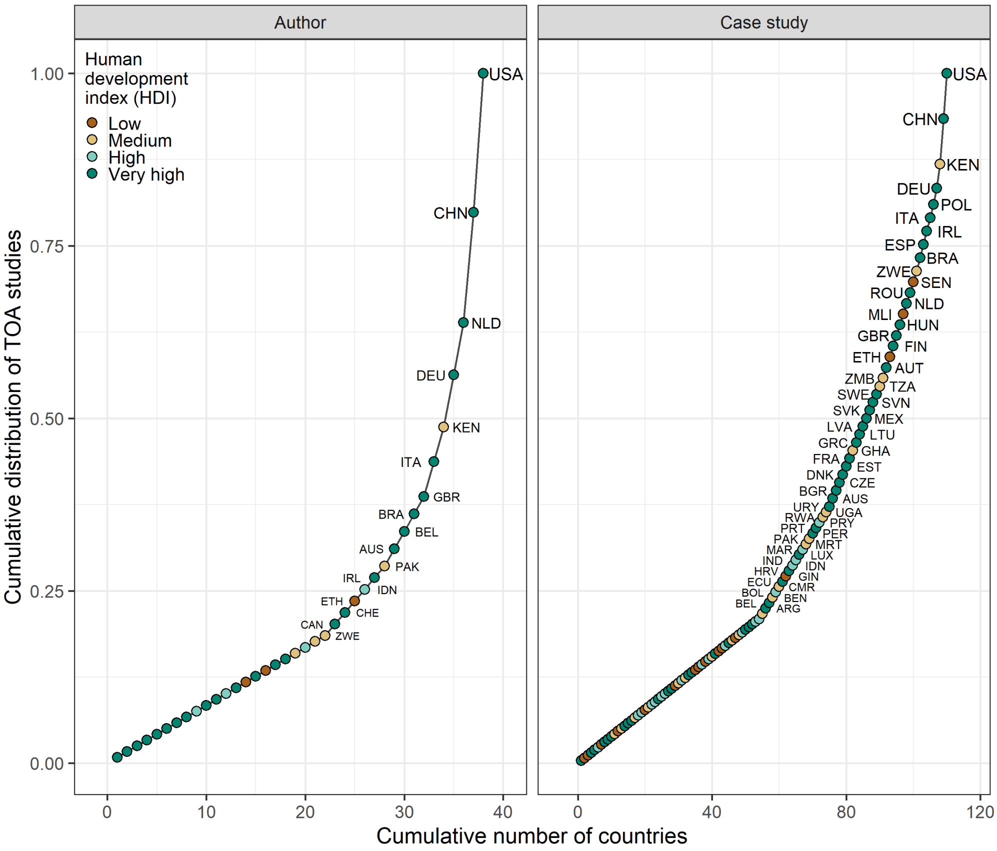

Figure 1: Cumulative distribution of TOA articles versus cumulative number of countries, both in terms of the corresponding author's affiliation and case-study area. Colors indicate the four classifications of the human development index (HDI) as provided by the UNDP for 2019.

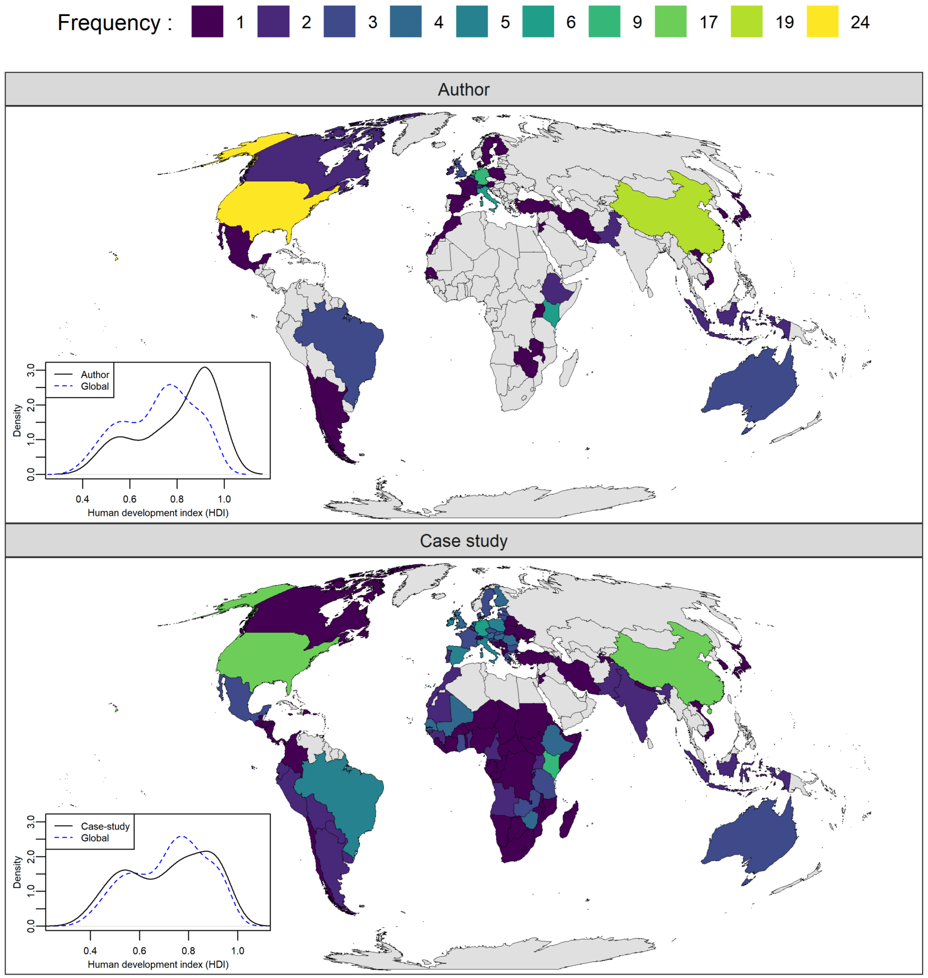

Figure 2: The frequency of a country as the location for the corresponding author's affiliation or the case-study area. Inset shows the density distributions of the human development index (HDI) for the author's affiliation and case-study area together with the density curves for all countries included in the HDI (Global).

2.2 Geographical trends of articles on TOA in agriculture

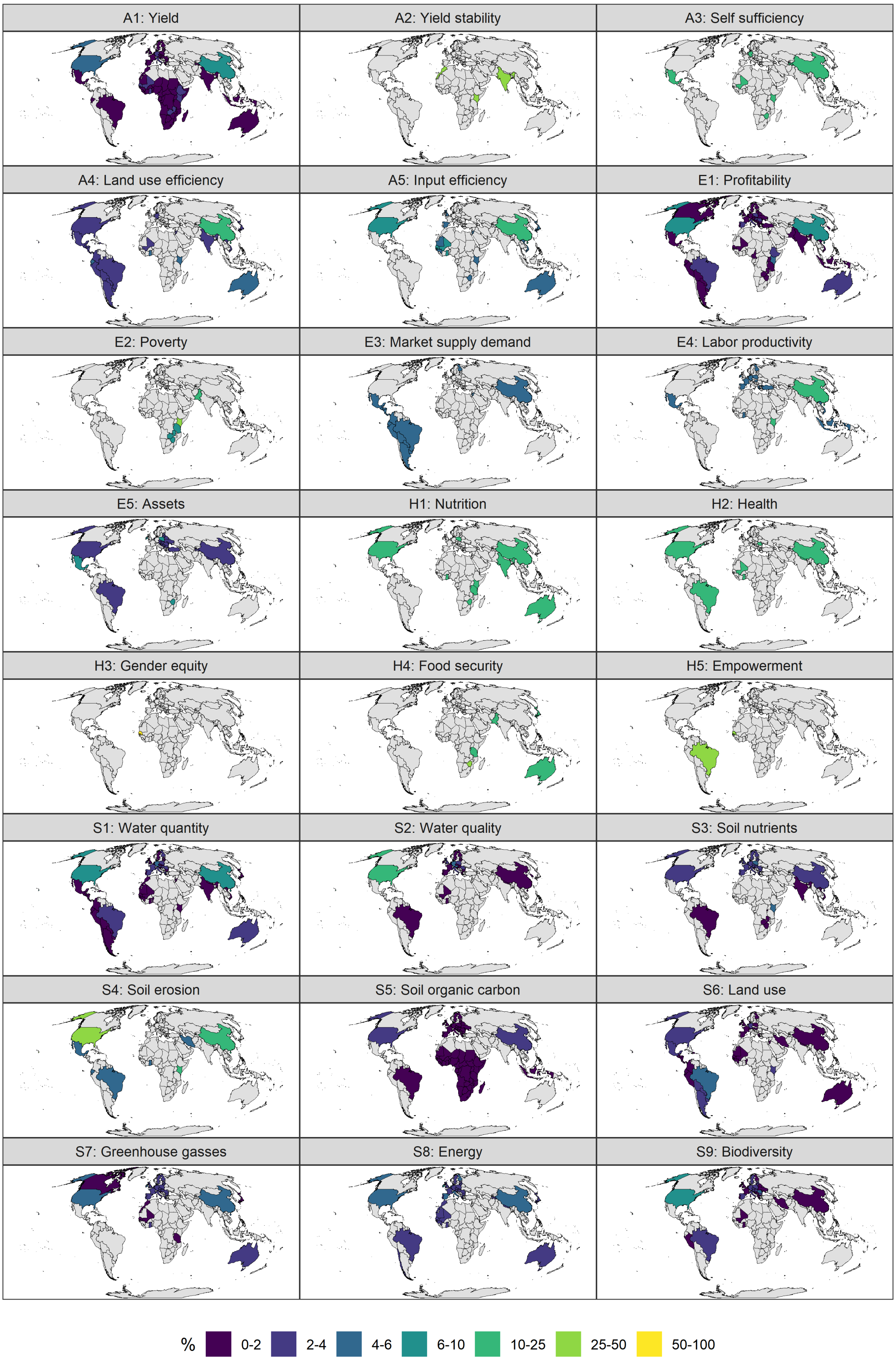

Figure 3: The relative frequency of a country in which the case-study area was located for each TOA indicator included within the article. The frequency of countries sums to 100% for each indicator panel.

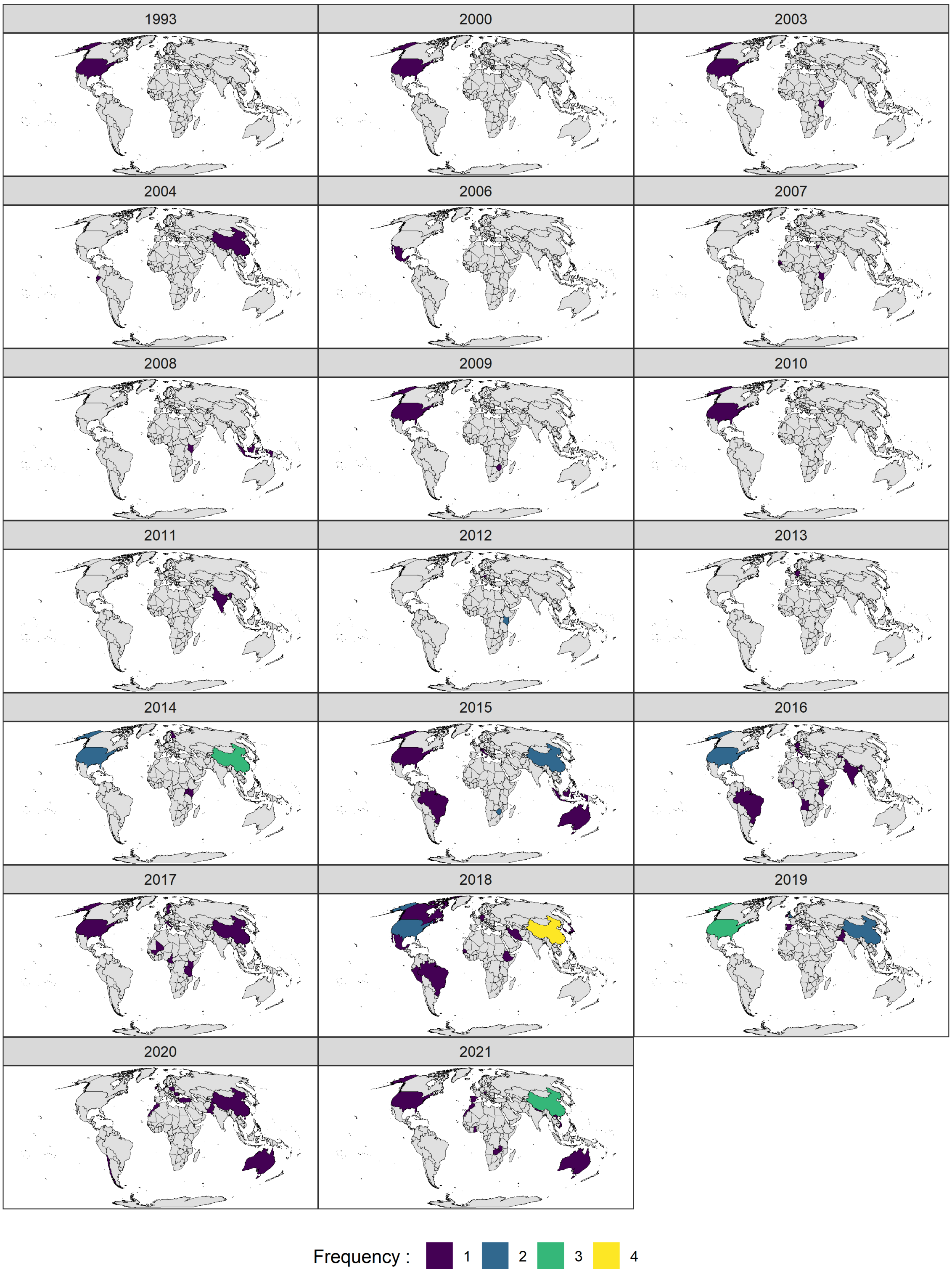

Figure 4: The frequency of a country in which the case-study area was located, plotted as a time series of publication date.

2.3 Cluster analysis

The cluster analysis (Fig. 1 in main manuscript) showed a clear distinction between articles that considered the TOA within an ecosystem services methodological framework (ESS). Results for this criterion were not reported in the main manuscript (see also the supplementary material included with the manuscript that contains the raw data).

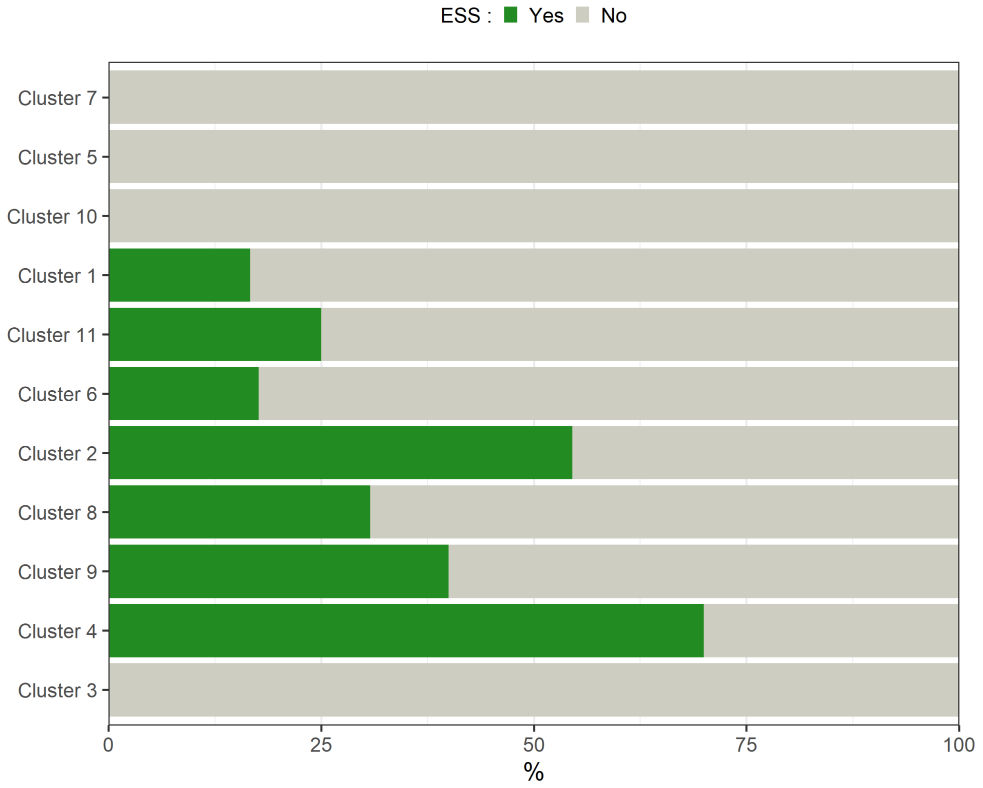

Figure 5: The frequency of whether an ecosystem services (ESS) methdological framework has been applied within an article, binned by each cluster

Fig. 6 shows the frequency of which system border was used to delineate the case-study area, binned by each cluster. Clusters that delineated the case-study area by administrative boundaries only (clusters 5 and 8) were characterized by economic, human health and agronomic indicators. Cluster 2 showed the highest share of biophysical delineation and was concerned with water quantity/quality, reflecting the use of watershed boundaries. Furthermore, 54 articles (43%) assessed biophysical indicators within their TOA but the case-study are was delineated by administrative boundaries only (not shown in graph).

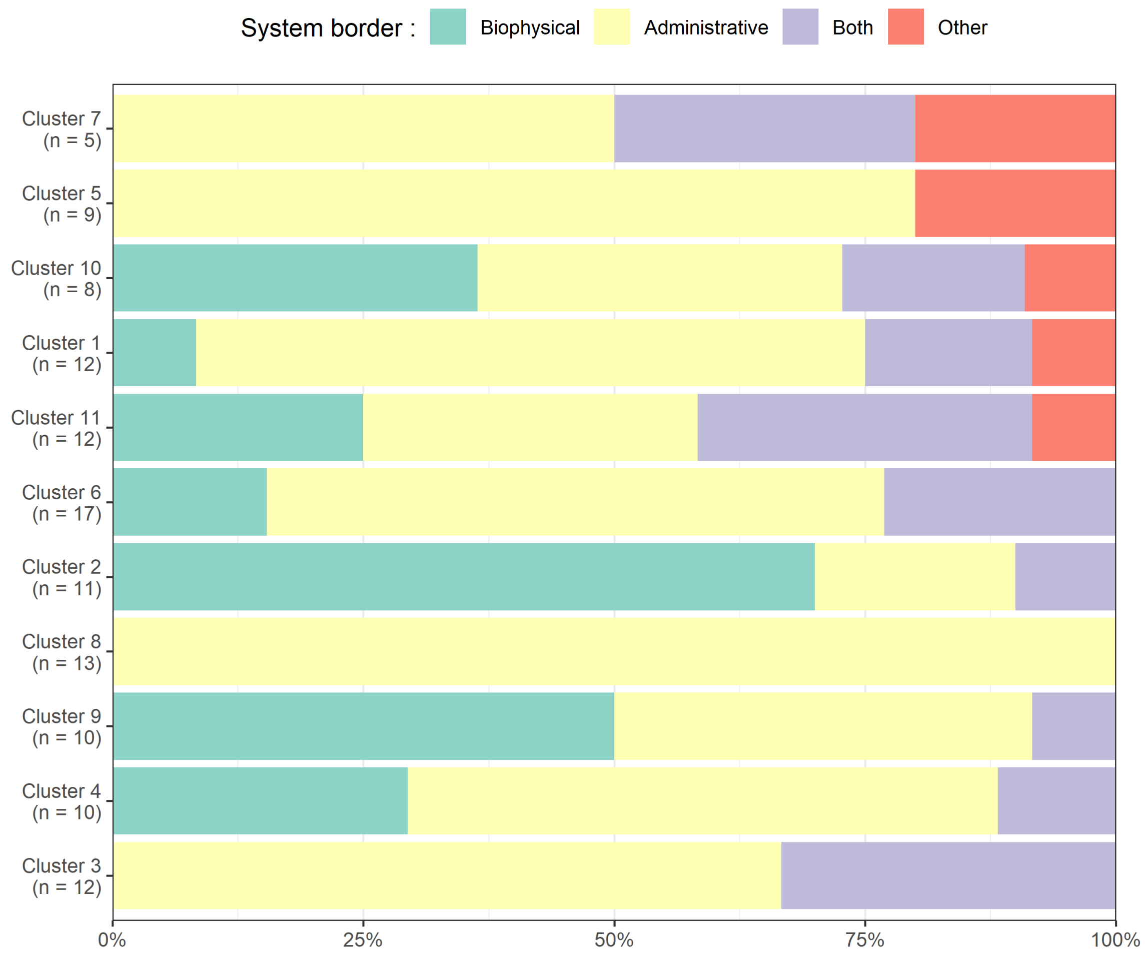

Figure 6: The frequency in which the TOA case-study area defined by administrative or biophysical borders for each cluster

2.4 Wordclouds

This section reports on wordclouds based on three qualitative criteria: farming system, farm management and reported knowledge gaps in the article's discussion section (see methodology main manuscript). Wordclouds have been generated by plotting words with a minimum frequency of three and a maximum of 100 words. Redundant words have been removed based on visual inspection.

One can infer from the wordcloud that 'small-holder' farms are the most prominent farming system studied, followed by 'livestock' and 'crop-livestock' production systems. This is not reflected within Fig. 3E in the main manuscript, where livestock took up a low share. This might show that the livestock component of the agricultural system under study is often disregarded or only accounted for implicitly. However, this wordlcoud is based on a smaller sample ($n = 81$) as not all articles explicitly listed the farming system under study. These words are followed by 'silviculture', 'semi-subsistence' and 'small-scale', mixed agriculture.

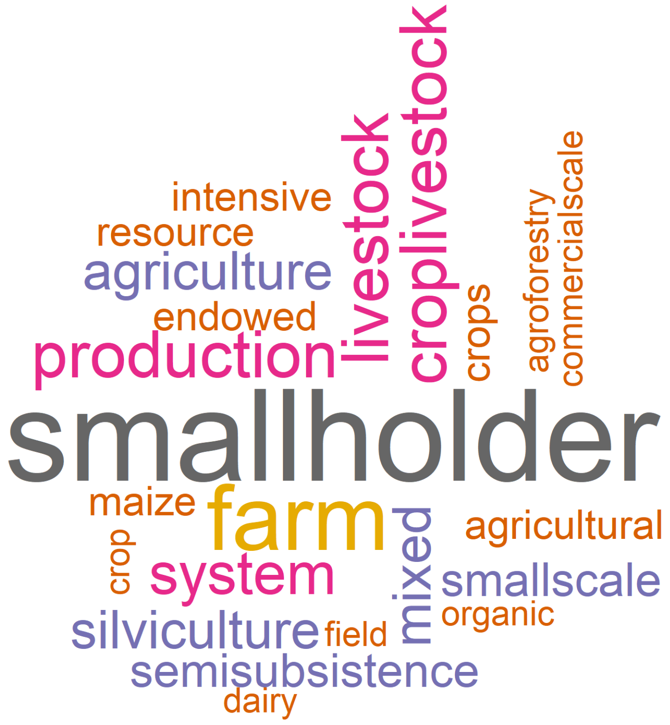

Figure 7: Word cloud for the "farming system" criterium (n=71)

The most prominent words are 'irrigated', 'maize' and 'wheat', followed by 'livestock', 'rainfed' and 'fertilizer'. 'Rice' is the third most common crop, together with 'corn', re-emphasizing the high frequency of articles that study maize/corn cropping systems. The third level (pink) lists words such as 'intensive', 'rotation', 'tillage', 'manure' and 'conservation', together with adjectives such as 'high', 'low', 'dry' and 'agricultural' (showing the limitation of wordclouds as these adjectives cannot be traced back to their respective nouns). Words in the fourth category show that 'organic' and 'inorganic' are listed in the same frequency. Notable are the words 'bioenergy' and 'vegetables', indicating niche farming systems relative to the overall trend. The last category lists management types such as 'intercropping', 'zero-grazing' and 'extensive' (note: less common in general although this should be ideally viewed in the context of the case-study area)

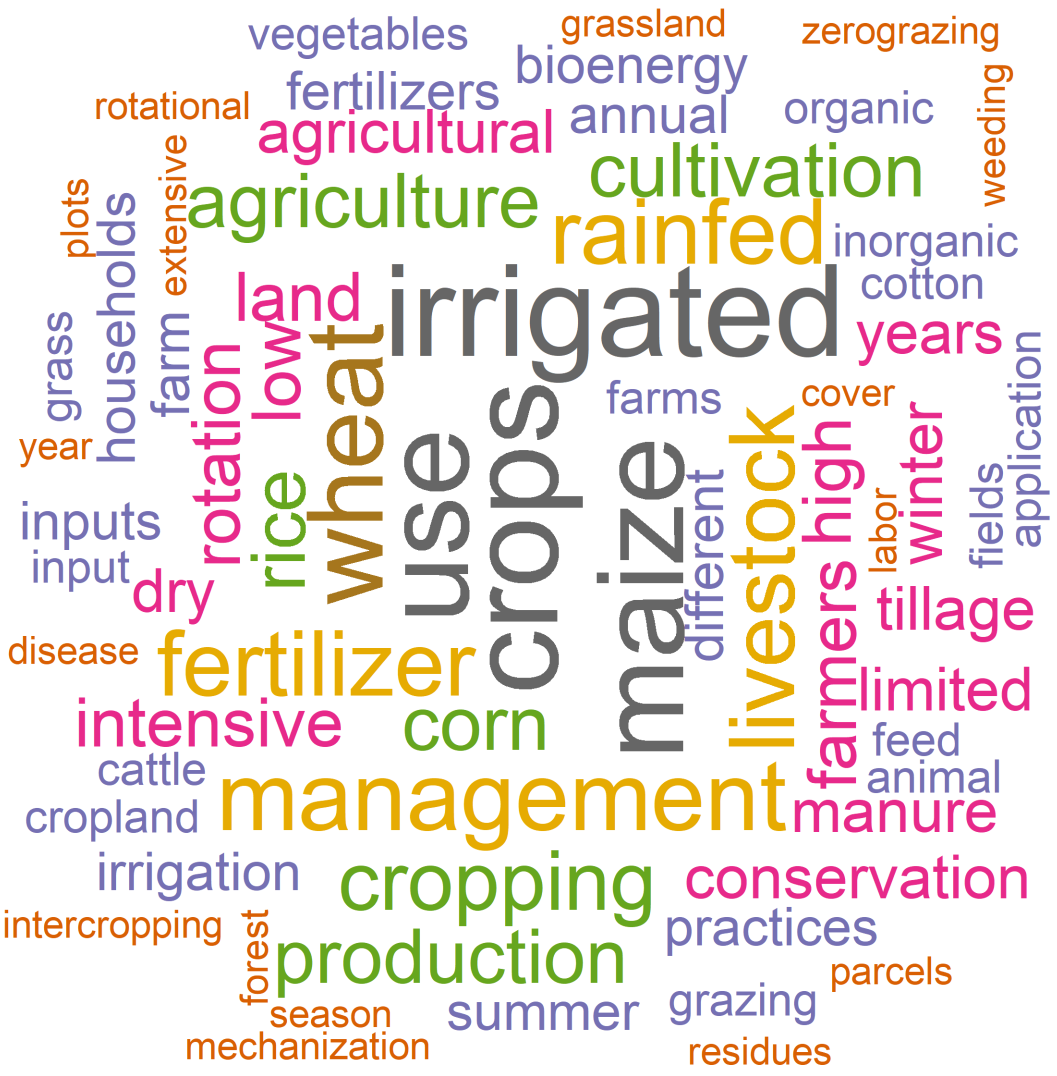

Figure 8: Word cloud for the "farm management" criterium (n=81)

A total of 99 articles from the sample reported knowledge gaps which have been visualized by a word cloud. The most prominent words are 'water' (26%) and 'ecosystem services' (24%). The latter indicates the frequency in which a need to include other ecosystem services is expressed in articles that adopt an ESS framework (24% of articles adopted an ESS framework).
The next two categories indicate the frequent mention that TOA should be performed for different crops (17—18%) (yellow category) and the words 'climate', 'economic' and 'management' (15—16% occurrence, in green). Words such as 'land use change', 'policy' and 'data' indicate that these factors need 'additional' study or be accounted for ('account') (10—13% occurrence).
The fourth category (purple) lists words relative to how the TOA framework is defined ('scale' (9%) and 'levels', 'multiple', 'objectives', 'inclusion' and 'stakeholders' occurring in 7% of the articles) as well as the study's robustness: 'uncertainty', 'constraints', 'required' (7—8%). With additional mention of TOA indicators ('GHG' (9%) and 'biodiversity', 'soil carbon', 'land use', 'yield' and 'market' occurring in 7% of the articles). It also lists 'impacts' (9%), 'decision-making' and 'performance' (7%), illustrating the need to use TOA results within an applied policy- and decision-making context.
Lastly, some words in orange include TOA indicators which showed a low frequency in Fig. 2A in the main manuscript: 'human' (6%) and 'labor', 'supply', 'nutrient' (4—5%) as well as two TOA indicators that showed a medium frequency in Fig. 2A in the main manuscript: 'species' (biodiversity) and 'energy' (4%).

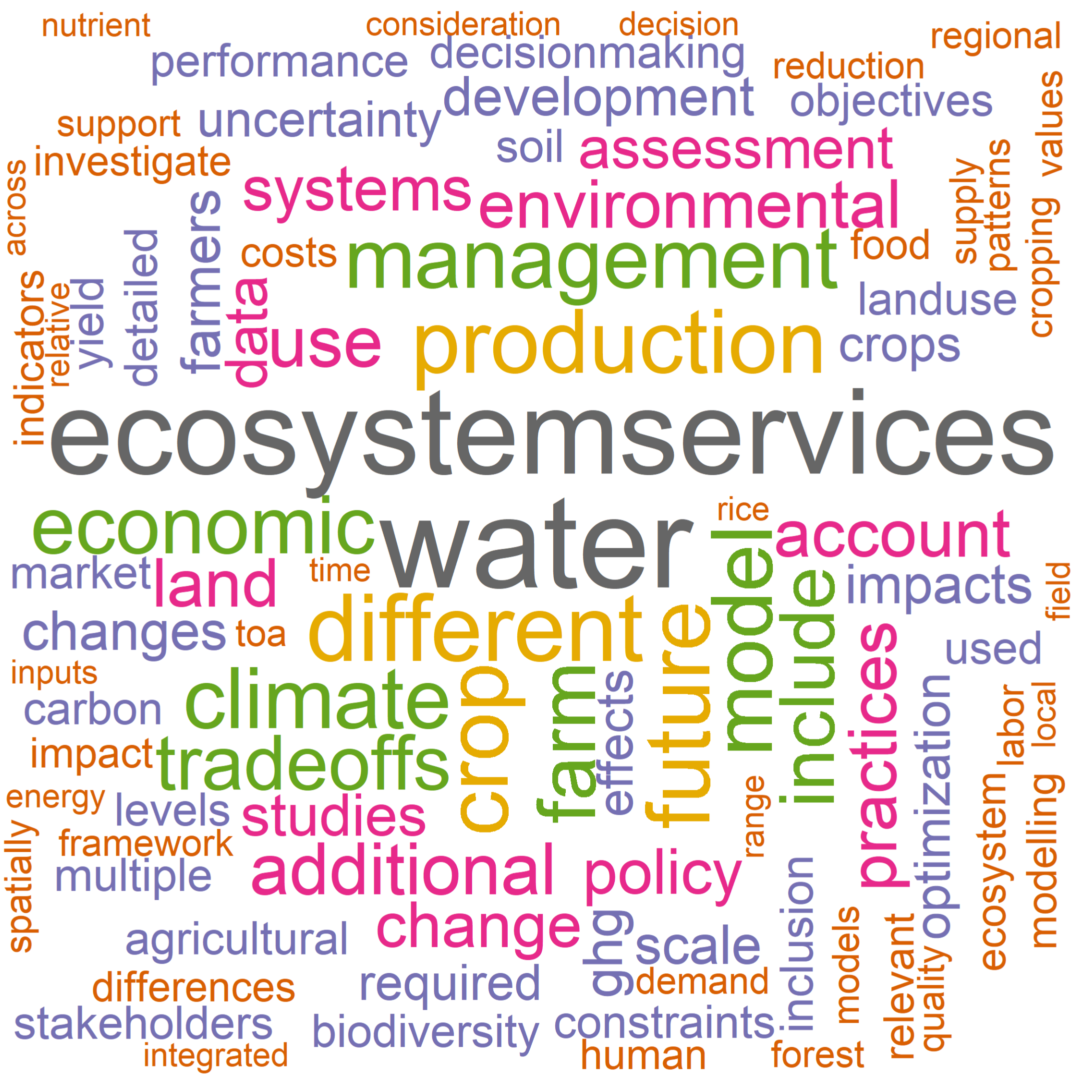

Figure 9: Word cloud for the "knowledge gaps" criterium (n=99)

3 Publications used in the systematic review

- Agrell, P. J., Stam, A., & Fischer, G. W. (2004). Interactive multiobjective agro-ecological land use planning: The Bungoma region in Kenya. European Journal of Operational Research, 158(1), 194-217. doi:10.1016/s0377-2217(03)00355-2
- Akhbari, M., & Grigg, N. S. (2014). Water Management Trade-offs between Agriculture and the Environment: A Multiobjective Approach and Application. Journal of Irrigation and Drainage Engineering, 140(8), 11. doi:10.1061/(asce)ir.1943-4774.0000737
- Al-Assaf, A., Salman, A. Z., Fisher, F. M., & Al-Karablieh, E. (2007). A trade-off analysis for the use of different water sources for irrigation (the case of Southern Shounah in the Jordan Valley). Water International, 32(2), 244-253. doi:10.1080/02508060708692204
- Albert, C., Hermes, J., Neuendorf, F., von Haaren, C., & Rode, M. (2016). Assessing and Governing Ecosystem Services Trade-Offs in Agrarian Landscapes: The Case of Biogas. Land, 5(1), 17. doi:10.3390/land5010001
- Anser, M. K., Hina, T., Hameed, S., Nasir, M. H., Ahmad, I., & Naseer, M. A. U. (2020). Modeling Adaptation Strategies against Climate Change Impacts in Integrated Rice-Wheat Agricultural Production System of Pakistan. International Journal of Environmental Research and Public Health, 17(7), 18. doi:10.3390/ijerph17072522
- Antle, J. M. (2019). DATA, ECONOMICS AND COMPUTATIONAL AGRICULTURAL SCIENCE. American Journal of Agricultural Economics, 101(2), 365-382. doi:10.1093/ajae/aay103
- Antle, J. M., Stoorvogel, J. J., & Valdivia, R. O. (2014). New parsimonious simulation methods and tools to assess future food and environmental security of farm populations. Philosophical Transactions of the Royal Society B-Biological Sciences, 369(1639), 15. doi:10.1098/rstb.2012.0280
- Armatas, C. A., Campbell, R. M., Watson, A. E., Borrie, W. T., Christensen, N., & Venn, T. J. (2018). An integrated approach to valuation and tradeoff analysis of ecosystem services for national forest decision-making. Ecosystem Services, 33, 1-18. doi:10.1016/j.ecoser.2018.07.007
- Asadolahi, Z., Salmanmahiny, A., Sakieh, Y., Mirkarimi, S. H., Baral, H., & Azimi, M. (2018). Dynamic trade-off analysis of multiple ecosystem services under land use change scenarios: Towards putting ecosystem services into planning in Iran. Ecological Complexity, 36, 250-260. doi:10.1016/j.ecocom.2018.09.003
- Baudron, F., Delmotte, S., Corbeels, M., Herrera, J. M., & Tittonell, P. (2015). Multi-scale trade-off analysis of cereal residue use for livestock feeding vs. soil mulching in the Mid-Zambezi Valley, Zimbabwe. Agricultural Systems, 134, 97-106. doi:10.1016/j.agsy.2014.03.002

- Bessette, D., Wilson, R., Beaudrie, C., & Schroeder, C. (2019). An online decision support tool to evaluate ecological weed management strategies. Weed Science, 67(4), 463-473. doi:10.1017/wsc.2019.21
- Bisseleua, D. H. B., Begoude, D., Tonnang, H., & Vidal, S. (2017). Ant-mediated ecosystem services and disservices on marketable yield in cocoa agroforestry systems. Agriculture Ecosystems & Environment, 247, 409-417. doi:10.1016/j.agee.2017.07.004
- Bostian, M., Whittaker, G., Barnhart, B., Fare, R., & Grosskopf, S. (2015). Valuing water quality tradeoffs at different spatial scales: An integrated approach using bilevel optimization. Water Resources and Economics, 11, 1-12. doi:10.1016/j.wre.2015.06.002
- Bostian, M. B., & Herlihy, A. T. (2014). Valuing tradeoffs between agricultural production and wetland condition in the U.S. Mid-Atlantic region. Ecological Economics, 105, 284-291. doi:10.1016/j.ecolecon.2014.06.016
- Braasch, M., Garcia-Barrios, L., Cortina-Villar, S., Huber-Sannwald, E., & Ramirez-Marcial, N. (2018). TRUE GRASP: Actors visualize and explore hidden limitations of an apparent win-win land management strategy in a MAB reserve. Environmental Modelling & Software, 105, 153-170. doi:10.1016/j.envsoft.2018.03.022
- Breen, M., Murphy, M. D., & Upton, J. (2019). Development of a dairy multi-objective optimization (DAIRYMOO) method for economic and environmental optimization of dairy farms. Applied Energy, 242, 1697-1711. doi:10.1016/j.apenergy.2019.03.059
- Breen, M., Upton, J., & Murphy, M. D. (2020). Photovoltaic systems on dairy farms: Financial and renewable multi-objective optimization (FARMOO) analysis. Applied Energy, 278, 14. doi:10.1016/j.apenergy.2020.115534
- Canan, S., & Ceyhan, V. (2020). The link between production efficiency and opportunity cost of protecting environment in TR83 region, Turkey. Environmental Science and Pollution Research, 27(28), 35112-35125. doi:10.1007/s11356-020-09726-9
- Celio, E., Ott, M., Siren, E., & Gret-Regamey, A. (2015). A prototypical tool for normative landscape scenario development and the analysis of actors' policy preferences. Landscape and Urban Planning, 137, 40-53. doi:10.1016/j.landurbplan.2014.12.013
- Claessens, L., Antle, J. M., Stoorvogel, J. J., Valdivia, R. O., Thornton, P. K., & Herrero, M. (2012). A method for evaluating climate change adaptation strategies for small-scale farmers using survey, experimental and modeled data. Agricultural Systems, 111, 85-95. doi:10.1016/j.agsy.2012.05.003
- Claessens, L., Stoorvogel, J. J., & Antle, J. M. (2008). Ex ante assessment of dual-purpose sweet potato in the crop-livestock system of western Kenya: A minimum-data approach. Agricultural Systems, 99(1), 13-22. doi:10.1016/j.agsy.2008.09.002

- Contant, C. K., Duffy, M. D., & Holub, M. A. (1993). DETERMINING TRADEOFFS BETWEEN WATER-QUALITY AND PROFITABILITY IN AGRICULTURAL PRODUCTION - IMPLICATIONS FOR NONPOINT-SOURCE POLLUTION POLICY. Water Science and Technology, 28(3-5), 27-34. Retrieved from ¡Go to ISI¿://WOS:A1993MA14800004

- DeFries, R., Mondal, P., Singh, D., Agrawal, I., Fanzo, J., Remans, R., & Wood, S. (2016). Synergies and trade-offs for sustainable agriculture: Nutritional yields and climate-resilience for cereal crops in Central India. Global Food Security-Agriculture Policy Economics and Environment, 11, 44-53. doi:10.1016/j.gfs.2016.07.001

- Devkota, M., Patil, S. B., Kumar, S., Kehel, Z., & Wery, J. (2021). Performance of elite genotypes of barley, chickpea, lentil, and wheat under conservation agriculture in Mediterranean rainfed conditions. Experimental Agriculture, 57(2), 126-143. doi:10.1017/s0014479721000107

- Diagana, B., Antle, J., Stoorvogel, J., & Gray, K. (2007). Economic potential for soil carbon sequestration in the Nioro region of Senegal's Peanut Basin. Agricultural Systems, 94(1), 26-37. doi:10.1016/j.agsy.2005.08.010

- Duku, C., Zwart, S. J., & Hein, L. (2016). Modelling the forest and woodland-irrigation nexus in tropical Africa: A case study in Benin. Agriculture Ecosystems & Environment, 230, 105-115. doi:10.1016/j.agee.2016.06.001

- Duval, D., & Colby, B. (2017). The influence of Colorado River flows on the upper Gulf of California fisheries economy. Ecological Engineering, 106, 791-798. doi:10.1016/j.ecoleng.2016.05.017

- Falconnier, G. N., Descheemaeker, K., Van Mourik, T. A., Adam, M., Sogoba, B., & Giller, K. E. (2017). Co-learning cycles to support the design of innovative farm systems in southern Mali. European Journal of Agronomy, 89, 61-74. doi:10.1016/j.eja.2017.06.008

- Fan, D. L., Fan, Y. D., Tian, Z., Li, X. B., Jiang, M., Sun, L. X., . . . Jiang, L. G. (2021). A vital option for food security and greenhouse gases mitigation: planting elite super rice in double- to single-rice cropping fields in China. Environmental Research Letters, 16(9), 13. doi:10.1088/1748-9326/ac1e3e

- Fu, Q. A., Zhu, C. H., Jiang, Q. X., Guo, H., & Zhao, K. (2015). Water resource management based on trade-off analysis of multi-dimensional critical regulation and control indicators. Water Science and Technology-Water Supply, 15(3), 552-558. doi:10.2166/ws.2015.004

- Giordano, R., Pluchinotta, I., Pagano, A., Scrieciu, A., & Nanu, F. (2020). Enhancing nature-based solutions acceptance through stakeholders' engagement in co-benefits identification and trade-offs analysis. Science of the Total Environment, 713, 18. doi:10.1016/j.scitotenv.2020.136552

- Gissi, E., Gaglio, M., Aschonitis, V. G., Fano, E. A., & Reho, M. (2018). Soil-related ecosystem services trade-off analysis for sustainable biodiesel production. Biomass & Bioenergy, 114, 83-99. doi:10.1016/j.biombioe.2017.08.028

- Gonzalez, J. M., Olivares, M. A., Medellin-Azuara, J., & Moreno, R. (2020). Multipurpose Reservoir Operation: a Multi-Scale Tradeoff Analysis between Hydropower Generation and Irrigated Agriculture. Water Resources Management, 34(9), 2837-2849. doi:10.1007/s11269-020-02586-5

- Gray, G. M., & Hammitt, J. K. (2000). Risk/risk trade-offs in pesticide regulation: An exploratory analysis of the public health effects of a ban on organophosphate and carbamate pesticides. Risk Analysis, 20(5), 665-680. doi:10.1111/0272-4332.205060

- Gutsch, M., Lasch-Born, P., Kollas, C., Suckow, F., & Reyer, C. P. O. (2018). Balancing trade-offs between ecosystem services in Germany's forests under climate change. Environmental Research Letters, 13(4), 12. doi:10.1088/1748-9326/aab4e5

- Habtemariam, L. T., Kassa, G. A., & Gandorfer, M. (2017). Impact of climate change on farms in smallholder farming systems: Yield impacts, economic implications and distributional effects. Agricultural Systems, 152, 58-66. doi:10.1016/j.agsy.2016.12.006

- Hayha, T., Franzese, P. P., Paletto, A., & Fath, B. D. (2015). Assessing, valuing, and mapping ecosystem services in Alpine forests. Ecosystem Services, 14, 12-23. doi:10.1016/j.ecoser.2015.03.001

- Herrera, B., Gerster-Bentaya, M., Tzouramani, I., & Knierim, A. (2019). Advisory services and farm-level sustainability profiles: an exploration in nine European countries. Journal of Agricultural Education & Extension, 25(2), 117-137. doi:10.1080/1389224x.2019.1583817

- Ho, T. Q., Hoang, V. N., & Wilson, C. (2021). Trade-off analysis of cost and nutrient efficiency of coffee farms in vietnam: A more generalised approach. Journal of Environmental Management, 279, 10. doi:10.1016/j.jenvman.2020.111601

- Hochman, Z., Horan, H., Garcia, J. N., Hopwood, G., Whish, J., Bell, L., . . . Jing, H. C. (2020). Cropping system yield gaps can be narrowed with more optimal rotations in dryland subtropical Australia. Agricultural Systems, 184, 9. doi:10.1016/j.agsy.2020.102896

- Huang, L. C., Chen, Y. H., Chen, Y. H., Wang, C. F., & Hu, M. C. (2018). Food-Energy Interactive Tradeoff Analysis of Sustainable Urban Plant Factory Production Systems. Sustainability, 10(2), 12. doi:10.3390/su10020446

- Ilukor, J., Bagamba, F., & Bashaasha, B. (2014). Application of the TOA-MD model to assess adoption potential of improved sweet potato technologies by rural poor farm households under climate change: the case of Kabale district in Uganda. Food Security, 6(3), 359-368. doi:10.1007/s12571-014-0350-8

- Jeon, D. J., Ki, S. J., Cha, Y., Park, Y., & Kim, J. H. (2018). New methodology of evaluation of best management practices performances for an agricultural watershed according to the climate change scenarios: A hybrid

use of deterministic and decision support models. Ecological Engineering, 119, 73-83. doi:10.1016/j.ecoleng.2018.05.006

- Karner, K., Schmid, E., Schneider, U. A., & Mitter, H. (2021). Computing stochastic Pareto frontiers between economic and environmental goals for a semi-arid agricultural production region in Austria. Ecological Economics, 185, 19. doi:10.1016/j.ecolecon.2021.107044

- Khasanah, N., Perdana, A., Rahmanullah, A., Manurung, G., Roshetko, J. M., & van Noordwijk, M. (2015). Intercropping teak (Tectona grandis) and maize (Zea mays): bioeconomic trade-off analysis of agroforestry management practices in Gunungkidul, West Java. Agroforestry Systems, 89(6), 1019-1033. doi:10.1007/s10457-015-9832-8

- Laurita, B., Castelli, G., Resta, C., & Bresci, E. (2021). Stakeholder-based water allocation modelling and ecosystem services trade-off analysis: the case of El Carracillo region (Spain). Hydrological Sciences Journal-Journal Des Sciences Hydrologiques, 66(5), 777-794. doi:10.1080/02626667.2021.1895439

- Lautenbach, S., Volk, M., Strauch, M., Whittaker, G., & Seppelt, R. (2013). Optimization-based trade-off analysis of biodiesel crop production for managing an agricultural catchment. Environmental Modelling & Software, 48, 98-112. doi:10.1016/j.envsoft.2013.06.006

- Lee, S. H., Assi, A. T., Daher, B., Mengoub, F. E., & Mohtar, R. H. (2020). A Water-Energy-Food Nexus approach for conducting trade-off analysis: Morocco's phosphate industry in the Khouribga region. Hydrology and Earth System Sciences, 24(10), 4727-4741. doi:10.5194/hess-24-4727-2020

- Lee, S. H., Taniguchi, M., Mohtar, R. H., Choi, J. Y., & Yoo, S. H. (2018). An Analysis of the Water-Energy-Food-Land Requirements and CO2 Emissions for Food Security of Rice in Japan. Sustainability, 10(9), 16. doi:10.3390/su10093354

- Li, S. B., Thompson, M., Moussavi, S., & Dvorak, B. (2021). Life cycle and economic assessment of corn production practices in the western US Corn Belt. Sustainable Production and Consumption, 27, 1762-1774. doi:10.1016/j.spc.2021.04.021

- Li, Z. C., Song, Z. L., Yang, X. M., Song, A. L., Yu, C. X., Wang, T., . . . Liang, Y. C. (2018). Impacts of silicon on biogeochemical cycles of carbon and nutrients in croplands. Journal of Integrative Agriculture, 17(10), 2182-2195. doi:10.1016/s2095-3119(18)62018-0

- Lu, C. H., & van Ittersum, M. K. (2004). A trade-off analysis of policy objectives for Ansai, the Loess Plateau of China. Agriculture Ecosystems & Environment, 102(3), 235-246. doi:10.1016/j.agee.2003.09.023

- Lu, Y. C., Teasdale, J. R., & Huang, W. Y. (2003). An economic and environmental tradeoff analysis of sustainable agriculture cropping systems. Journal of Sustainable Agriculture, 22(3), 25-41. doi:10.1300/J064v22n03$_0$5

- Lu, Z. X., Wei, Y. P., Xiao, H. L., Zou, S. B., Ren, J., & Lyle, C. (2015). Trade-offs between midstream agricultural production and downstream ecological sustainability in the Heihe River basin in the past half century. Agricultural Water Management, 152, 233-242. doi:10.1016/j.agwat.2015.01.022

- Makkonen, M., Huttunen, S., Primmer, E., Repo, A., & Hilden, M. (2015). Policy coherence in climate change mitigation: An ecosystem service approach to forests as carbon sinks and bioenergy sources. Forest Policy and Economics, 50, 153-162. doi:10.1016/j.forpol.2014.09.003

- Maraseni, T., An-Vo, D. A., Mushtaq, S., & Reardon-Smith, K. (2021). Carbon smart agriculture: An integrated regional approach offers significant potential to increase profit and resource use efficiency, and reduce emissions. Journal of Cleaner Production, 282, 14. doi:10.1016/j.jclepro.2020.124555

- Masikati, P., Sisito, G., Chipatela, F., Tembo, H., & Winowiecki, L. A. Agriculture extensification and associated socio-ecological trade-offs in smallholder farming systems of Zambia. International Journal of Agricultural Sustainability, 12. doi:10.1080/14735903.2021.1907108

- Mendes, L. A., de Barros, M. T. L., Zambon, R. C., & Yeh, W. W. G. (2015). Trade-Off Analysis among Multiple Water Uses in a Hydropower System: Case of Sao Francisco River Basin, Brazil. Journal of Water Resources Planning and Management, 141(10), 10. doi:10.1061/(asce)wr.1943-5452.0000527

- Modongo, O., & Kulshreshtha, S. N. (2018). Economics of mitigating greenhouse gas emissions from beef production in western Canada. Agricultural Systems, 162, 229-238. doi:10.1016/j.agsy.2017.12.008

- Mulwa, R., Rao, K. P. C., Gummadi, S., & Kilavi, M. (2016). Impacts of climate change on agricultural household welfare in Kenya. Climate Research, 67(2), 87-97. doi:10.3354/cr01357

- Mushtaq, S., Maraseni, T. N., Reardon-Smith, K., Bundschuh, J., & Jackson, T. (2015). Integrated assessment of water-energy-GHG emissions tradeoffs in an irrigated lucerne production system in eastern Australia. Journal of Cleaner Production, 103, 491-498. doi:10.1016/j.jclepro.2014.05.037

- Mwambo, F. M., Furst, C., Martius, C., Jimenez-Martinez, M., Nyarko, B. K., & Borgemeister, C. (2021). Combined application of the EM-DEA and EX-ACT approaches for integrated assessment of resource use efficiency, sustainability and carbon footprint of smallholder maize production practices in sub-Saharan Africa. Journal of Cleaner Production, 302, 19. doi:10.1016/j.jclepro.2021.126132

- Naqvi, S. A. A., Nadeem, A. M., Iqbal, M. A., Ali, S., & Naseem, A. (2019). Assessing the Vulnerabilities of Current and Future Production Systems in Punjab, Pakistan. Sustainability, 11(19), 13. doi:10.3390/su11195365

- Nguyen, T. H., Cook, M., Field, J. L., Khuc, Q. V., & Paustian, K. (2018). High-resolution trade-off analysis and optimization of ecosystem services and disservices in agricultural landscapes. Environmental Modelling & Software, 107, 105-118. doi:10.1016/j.envsoft.2018.06.006

- Nguyen, T. H., Granger, J., Pandya, D., & Paustian, K. (2019). High-resolution multi-objective optimization of feedstock landscape design for hybrid first and second generation biorefineries. Applied Energy, 238, 1484-1496. doi:10.1016/j.apenergy.2019.01.117

- Nie, Y. L., Avraamidou, S., Xiao, X., Pistikopoulos, E. N., Li, J., Zeng, Y. J., . . . Zhu, M. (2019). A Food-Energy-Water Nexus approach for land use optimization. Science of the Total Environment, 659, 7-19. doi:10.1016/j.scitotenv.2018.12.242

- Oleson, K. L. L., Falinski, K. A., Lecky, J., Rowe, C., Kappel, C. V., Selkoe, K. A., & White, C. (2017). Upstream solutions to coral reef conservation: The payoffs of smart and cooperative decision-making. Journal of Environmental Management, 191, 8-18. doi:10.1016/j.jenvman.2016.12.067

- Pakhtigian, E. L., Jeuland, M., Bharati, L., & Pandey, V. P. (2021). The role of hydropower in visions of water resources development for rivers of Western Nepal. International Journal of Water Resources Development, 37(3), 531-558. doi:10.1080/07900627.2019.1600474

- Pang, A. P., Li, C. H., Sun, T., Yang, W., & Yang, Z. F. (2018). Trade-Off Analysis to Determine Environmental Flows in a Highly Regulated Watershed. Scientific Reports, 8, 11. doi:10.1038/s41598-018-32126-6

- Pang, A. P., & Sun, T. (2014). Bayesian networks for environmental flow decision-making and an application in the Yellow River estuary, China. Hydrology and Earth System Sciences, 18(5), 1641-1651. doi:10.5194/hess-18-1641-2014

- Pang, A. P., Zhao, F., Li, C. H., & Yi, Y. J. (2021). Rethinking Environmental Flows for the Yellow River Estuary by Trading Off Crop Yield and Ecological Benefits. Agriculture-Basel, 11(2), 16. doi:10.3390/agriculture11020116

- Pang, X., Mortberg, U., Sallnas, O., Trubins, R., Nordstrom, E. M., & Bottcher, H. (2017). Habitat network assessment of forest bioenergy options using the landscape simulator LandSim - A case study of Kronoberg, southern Sweden. Ecological Modelling, 345, 99-112. doi:10.1016/j.ecolmodel.2016.12.006

- Picchi, P., Verzandvoort, S., Geneletti, D., Hendriks, K., & Stremke, S. Deploying ecosystem services to develop sustainable energy landscapes: a case study from the Netherlands. Smart and Sustainable Built Environment, 16. doi:10.1108/sasbe-02-2020-0010

- Popp, A., Dietrich, J. P., Lotze-Campen, H., Klein, D., Bauer, N., Krause, M., . . . Edenhofer, O. (2011). The economic potential of bioenergy for climate change mitigation with special attention given to implications for the land system. Environmental Research Letters, 6(3), 9. doi:10.1088/1748-9326/6/3/034017

- Prestele, R., & Verburg, P. H. (2020). The overlooked spatial dimension of climate-smart agriculture. Global Change Biology, 26(3), 1045-1054. doi:10.1111/gcb.14940

- Randhir, T. O., & Tsvetkova, O. (2009). Watershed-Scale Tradeoffs in Water Quantity and Quality Attributes for Conservation Policy. Water Air and Soil Pollution, 201(1-4), 347-363. doi:10.1007/s11270-008-9949-8

- Recanati, F., & Guariso, G. (2018). An optimization model for the planning of agroecosystems: Trading off socio-economic feasibility and biodiversity. Ecological Engineering, 117, 194-204. doi:10.1016/j.ecoleng.2018.03.010

- Rodrigues, G. S., Martins, C. R., & de Barros, I. (2018). Sustainability assessment of ecological intensification practices in coconut production. Agricultural Systems, 165, 71-84. doi:10.1016/j.agsy.2018.06.001

- Roffeis, M., Fitches, E. C., Wakefield, M. E., Almeida, J., Valada, T. R. A., Devic, E., . . . Muys, B. (2020). Ex-ante life cycle impact assessment of insect based feed production in West Africa. Agricultural Systems, 178, 21. doi:10.1016/j.agsy.2019.102710

- Ruijs, A., Wossink, A., Kortelainen, M., Alkemade, R., & Schulp, C. J. E. (2013). Trade-off analysis of ecosystem services in Eastern Europe. Ecosystem Services, 4, 82-94. doi:10.1016/j.ecoser.2013.04.002

- Rukundo, E., Liu, S. L., Dong, Y. H., Rutebuka, E., Asamoah, E. F., Xu, J. W., & Wu, X. (2018). Spatio-temporal dynamics of critical ecosystem services in response to agricultural expansion in Rwanda, East Africa. Ecological Indicators, 89, 696-705. doi:10.1016/j.ecolind.2018.02.032

- Ruppen, S., Wolfgramm, B., Scheidegger, R., & Bader, H. P. (2016). Method for Analyzing Trade-offs in Biomass Management in Smallholder Farming Systems Based on Mass Balance. Mountain Research and Development, 36(1), 80-90. doi:10.1659/mrd-journal-d-14-00114.1

- Sacchelli, S., Garegnani, G., Geri, F., Grilli, G., Paletto, A., Zambelli, P., . . . Vettorato, D. (2016). Trade-off between photovoltaic systems installation and agricultural practices on arable lands: An environmental and socio-economic impact analysis for Italy. Land Use Policy, 56, 90-99. doi:10.1016/j.landusepol.2016.04.024

- Salmon, G., Teufel, N., Baltenweck, I., van Wijk, M., Claessens, L., & Marshall, K. (2018). Trade-offs in livestock development at farm level: Different actors with different objectives. Global Food Security-Agriculture Policy Economics and Environment, 17, 103-112. doi:10.1016/j.gfs.2018.04.002

- Sanon, S., Hein, T., Douven, W., & Winkler, P. (2012). Quantifying ecosystem service trade-offs: The case of an urban floodplain in Vienna, Austria. Journal of Environmental Management, 111, 159-172. doi:10.1016/j.jenvman.2012.06.008

- Schneibel, A., Stellmes, M., Roder, A., Finckh, M., Revermann, R., Frantz, D., & Hill, J. (2016). Evaluating the trade-off between food and timber resulting from the conversion of Miombo forests to agricultural land in Angola using multi-temporal Landsat data. Science of the Total Environment, 548, 390-401. doi:10.1016/j.scitotenv.2015.12.137

- Senthilkumar, K., Lubbers, M., de Ridder, N., Bindraban, P. S., Thiyagarajan, T. M., & Giller, K. E. (2011). Policies to support economic and environmental goals at farm and regional scales: Outcomes for rice farmers in Southern India depend on their resource endowment. Agricultural Systems, 104(1), 82-93. doi:10.1016/j.agsy.2010.10.001

- Shikuku, K. M., Valdivia, R. O., Paul, B. K., Mwongera, C., Winowiecki, L., Laderach, P., . . . Silvestri, S. (2017). Prioritizing climate-smart livestock technologies in rural Tanzania: A minimum data approach. Agricultural Systems, 151, 204-216. doi:10.1016/j.agsy.2016.06.004
- Sida, T. S., Baudron, F., Hadgu, K., Derero, A., & Giller, K. E. (2018). Crop vs. tree: Can agronomic management reduce trade-offs in tree-crop interactions? Agriculture Ecosystems & Environment, 260, 36-46. doi:10.1016/j.agee.2018.03.011
- Sieber, S., Amjath-Babu, T. S., Jansson, T., Muller, K., Tscherning, K., Graef, F., . . . Paloma, S. G. Y. (2013). Sustainability impact assessment using integrated meta-modelling: Simulating the reduction of direct support under the EU common agricultural policy (CAP). Land Use Policy, 33, 235-245. doi:10.1016/j.landusepol.2013.01.002
- Smukler, S. M., Sanchez-Moreno, S., Fonte, S. J., Ferris, H., Klonsky, K., O'Geen, A. T., . . . Jackson, L. E. (2010). Biodiversity and multiple ecosystem functions in an organic farmscape. Agriculture Ecosystems & Environment, 139(1-2), 80-97. doi:10.1016/j.agee.2010.07.004
- Speelman, E. N., Astier, M., Lopez-Ridaura, S., Leffelaar, P. A., & van Ittersum, M. K. (2006). Trade-off analysis for sustainability evaluation: a case study of the Purhepecha region, Mexico. Outlook on Agriculture, 35(1), 57-64. doi:10.5367/000000006776207609
- Stefanes, M., Ochoa-Quintero, J. M., Roque, F. D. O., Sugai, L. S. M., Tambosi, L. R., Lourival, R., & Laurance, S. (2016). Incorporating resilience and cost in ecological restoration strategies at landscape scale. Ecology and Society, 21(4), 11. doi:10.5751/es-08922-210454
- Stoorvogel, J. J., Antle, J. M., & Crissman, C. C. (2004). Trade-off analysis in the Northern Andes to study the dynamics in agricultural land use. Journal of Environmental Management, 72(1-2), 23-33. doi:10.1016/j.jenvman.2004.03.012
- Stosch, K. C., Quilliam, R. S., Bunnefeld, N., & Oliver, D. M. (2019). Quantifying stakeholder understanding of an ecosystem service trade-off. Science of the Total Environment, 651, 2524-2534. doi:10.1016/j.scitotenv.2018.10.090
- Sylla, M., Hagemann, N., & Szewranski, S. (2020). Mapping trade-offs and synergies among peri-urban ecosystem services to address spatial policy. Environmental Science & Policy, 112, 79-90. doi:10.1016/j.envsci.2020.06.002
- Tayyebi, A., Tayyebi, A., Vaz, E., Arsanjani, J. J., & Helbich, M. (2016). Analyzing crop change scenario with the SmartScape (TM) spatial decision support system. Land Use Policy, 51, 41-53. doi:10.1016/j.landusepol.2015.11.002
- Tian, Z., Niu, Y. L., Fan, D. L., Sun, L. X., Ficsher, G., Zhong, H. L., . . . Tubiello, F. N. (2018). Maintaining rice production while mitigating methane and nitrous oxide emissions from paddy fields in China: Evaluating tradeoffs by using coupled agricultural systems models. Agricultural Systems, 159, 175-186. doi:10.1016/j.agsy.2017.04.006

- Tilmant, A., Pina, J., Salman, M., Casarotto, C., Ledbi, F., & Pek, E. (2020). Probabilistic trade-off assessment between competing and vulnerable water users - The case of the Senegal River basin. Journal of Hydrology, 587, 15. doi:10.1016/j.jhydrol.2020.124915
- Tittonell, P., Gerard, B., & Erenstein, O. (2015). Tradeoffs around crop residue biomass in smallholder crop-livestock systems - What's next? Agricultural Systems, 134, 119-128. doi:10.1016/j.agsy.2015.02.003
- Tittonell, P., van Wijk, M. T., Rufino, M. C., Vrugt, J. A., & Giller, K. E. (2007). Analysing trade-offs in resource and labour allocation by smallholder farmers using inverse modelling techniques: A case-study from Kakamega district, western Kenya. Agricultural Systems, 95(1-3), 76-95. doi:10.1016/j.agsy.2007.04.002
- Todman, L. C., Coleman, K., Milne, A. E., Gil, J. D. B., Reidsma, P., Schwoob, M. H., . . . Whitmore, A. P. (2019). Multi-objective optimization as a tool to identify possibilities for future agricultural landscapes. Science of the Total Environment, 687, 535-545. doi:10.1016/j.scitotenv.2019.06.070
- Tui, S. H. K., Valbuena, D., Masikati, P., Descheemaeker, K., Nyamangara, J., Claessens, L., . . . Nkomboni, D. (2015). Economic trade-offs of biomass use in crop-livestock systems: Exploring more sustainable options in semi-arid Zimbabwe. Agricultural Systems, 134, 48-60. doi:10.1016/j.agsy.2014.06.009
- Valdivia, R. O., Antle, J. M., & Stoorvogel, J. J. (2012). Coupling the Tradeoff Analysis Model with a market equilibrium model to analyze economic and environmental outcomes of agricultural production systems. Agricultural Systems, 110, 17-29. doi:10.1016/j.agsy.2012.03.003
- Valdivia, R. O., Antle, J. M., & Stoorvogel, J. J. (2017). Designing and evaluating sustainable development pathways for semi-subsistence crop-livestock systems: lessons from Kenya. Agricultural Economics, 48, 11-26. doi:10.1111/agec.12383
- van Noordwijk, M., Suyamto, D. A., Lusiana, B., Ekadinata, A., & Hairiah, K. (2008). Facilitating agroforestation of landscapes for sustainable benefits: Tradeoffs between carbon stocks and local development benefits in Indonesia according to the FALLOW model. Agriculture Ecosystems & Environment, 126(1-2), 98-112. doi:10.1016/j.agee.2008.01.016
- Viglizzo, E. F., & Frank, F. C. (2006). Land-use options for Del Plata Basin in South America: Tradeoffs analysis based on ecosystem service provision. Ecological Economics, 57(1), 140-151. doi:10.1016/j.ecolecon.2005.03.025
- Villalba, D., Diez-Unquera, B., Carrascal, A., Bernues, A., & Ruiz, R. (2019). Multi-objective simulation and optimisation of dairy sheep farms: Exploring trade-offs between economic and environmental outcomes. Agricultural Systems, 173, 107-118. doi:10.1016/j.agsy.2019.01.011
- Vogdrup-Schmidt, M., Strange, N., Olsen, S. B., & Thorsen, B. J. (2017). Trade-off analysis of ecosystem service provision in nature networks. Ecosystem Services, 23, 165-173. doi:10.1016/j.ecoser.2016.12.011

- Vrebos, D., Jones, A., Lugato, E., O'Sullivan, L., Schulte, R., Staes, J., & Meire, P. (2021). Spatial evaluation and trade-off analysis of soil functions through Bayesian networks. European Journal of Soil Science, 72(4), 1575-1589. doi:10.1111/ejss.13039

- Wan, N. F., Chen, J. Q., Jiang, J. X., & Li, B. (2017). A conceptual framework for ecosystem management based on tradeoff analysis. Ecological Indicators, 75, 352-361. doi:10.1016/j.ecolind.2016.12.032

- Winowiecki, L. A., Bourne, M., Magaju, C., Neely, C., Massawe, B., Masikati, P., . . . Sinclair, F. Bringing evidence to bear for negotiating tradeoffs in sustainable agricultural intensification using a structured stakeholder engagement process. International Journal of Agricultural Sustainability, 23. doi:10.1080/14735903.2021.1897297

- Wu, X. T., Wang, S., Fu, B. J., Liu, Y., & Zhu, Y. (2018). Land use optimization based on ecosystem service assessment: A case study in the Yanhe watershed. Land Use Policy, 72, 303-312. doi:10.1016/j.landusepol.2018.01.003

- Xu, E. Q., Zhang, H. Q., Yang, Y., & Zhang, Y. (2014). Integrating a Spatially Explicit Tradeoff Analysis for Sustainable Land Use Optimal Allocation. Sustainability, 6(12), 8909-8930. doi:10.3390/su6128909

- Xu, X. B., Liu, J. P., Tan, Y., & Yang, G. S. (2021). Quantifying and optimizing agroecosystem services in China's Taihu Lake Basin. Journal of Environmental Management, 277, 16. doi:10.1016/j.jenvman.2020.111440

- Xu, Y., Huang, G. H., & Shao, L. G. (2014). Agricultural farming planning and water resources management under fuzzy uncertainty. Engineering Optimization, 46(2), 270-288. doi:10.1080/0305215x.2013.768239

- Zhong, H. L., Feng, K. S., Sun, L. X., Tian, Z., Fischer, G., Cheng, L., & Castillo, R. M. (2021). Water-land tradeoffs to meet future demands for sugar crops in Latin America and the Caribbean: A bio-physical and socio-economic nexus perspective. Resources Conservation and Recycling, 169, 11. doi:10.1016/j.resconrec.2021.105510

- Zhu, E. Y., Deng, J. S., Wang, H. Q., Wang, K., Huang, L. Y., Zhu, G. J., . . . Shahtahmassebi, A. (2019). Identify the optimization strategy of nitrogen fertilization level based on trade-off analysis between rice production and greenhouse gas emission. Journal of Cleaner Production, 239, 11. doi:10.1016/j.jclepro.2019.118060

- Zingore, S., Gonzalez-Estrada, E., Delve, R. J., Herrero, M., Dimes, J. P., & Giller, K. E. (2009). An integrated evaluation of strategies for enhancing productivity and profitability of resource-constrained smallholder farms in Zimbabwe. Agricultural Systems, 101(1-2), 57-68. doi:10.1016/j.agsy.2009.03.003

4 References of the supplementary material

- Becker, R.A., Wilks, A.R.: original S code, Brownrigg, R. original R version. Enhancements by Thomas P Minka and Alex Deckmyn.

(2018). maps: Draw Geographical Maps. R package version 3.3.0. https://CRAN.R-project.org/package=maps.

- Fellows, I. (2018). worcloud: Word Clouds. R package version 2.6. https://CRAN.R-project.org/package=wordcloud.

- Wickham, H. (2021). forcats: Tools for Working with Categorical Variables (Factors). R package version 0.5.1. https://CRAN.R-project.org/package=forcats.

- Wickham, H. (2007). Reshaping Data with the reshape Package. Journal of Statistical Software, 21(12), 1-20. http://www.jstatsoft.org/v21/i12/.

- Wickham, H and Bryan, J (2019). readxl: Read Excel Files. R package version 1.3.1. https://CRAN.R-project.org/package=readxl.

- Wickham, H. and Seidel, D. (2020). scales: Scale Functions for Visualization. R package version 1.1.1. https://CRAN.R-project.org/package=scales.
