# Supplementary material for: A systematic review of the methodology of trade-off analysis in agriculture
Source: Nat Food. 2024 Mar 5;5(3):211–20. doi: 10.1038/s43016-024-00926-x (PMC10963264; doi:10.1038/s43016-024-00926-x)
Supplement: Supplementary file 2 — Reporting Summary [file 43016_2024_926_MOESM2_ESM.pdf]

nature research

Corresponding author(s): JCJ Groot, jeroen.groot@wur.nl

Last updated by author(s): Feb 14, 2024

Reporting Summary

Nature Research wishes to improve the reproducibility of the work that we publish. This form provides structure for consistency and transparency in reporting. For further information on Nature Research policies, see our Editorial Policies and the Editorial Policy Checklist.

Statistics

For all statistical analyses, confirm that the following items are present in the figure legend, table legend, main text, or Methods section.

n/a	Confirmed	
☐	☒	The exact sample size (*n*) for each experimental group/condition, given as a discrete number and unit of measurement
☒	☐	A statement on whether measurements were taken from distinct samples or whether the same sample was measured repeatedly
☒	☐	The statistical test(s) used AND whether they are one- or two-sided<br>*Only common tests should be described solely by name; describe more complex techniques in the Methods section.*
☒	☐	A description of all covariates tested
☐	☒	A description of any assumptions or corrections, such as tests of normality and adjustment for multiple comparisons
☒	☐	A full description of the statistical parameters including central tendency (e.g. means) or other basic estimates (e.g. regression coefficient) AND variation (e.g. standard deviation) or associated estimates of uncertainty (e.g. confidence intervals)
☒	☐	For null hypothesis testing, the test statistic (e.g. *F*, *t*, *r*) with confidence intervals, effect sizes, degrees of freedom and *P* value noted<br>*Give P values as exact values whenever suitable.*
☒	☐	For Bayesian analysis, information on the choice of priors and Markov chain Monte Carlo settings
☒	☐	For hierarchical and complex designs, identification of the appropriate level for tests and full reporting of outcomes
☒	☐	Estimates of effect sizes (e.g. Cohen's *d*, Pearson's *r*), indicating how they were calculated

Our web collection on statistics for biologists contains articles on many of the points above.

Software and code

Policy information about availability of computer code

Data collection	Criteria were logged within a Microsoft Office Excel (2021) sheet which has been made available under as a supplementary dataset.
Data analysis	The data collected during the systematic review has been further analyzed and visualized in R. Data handling, visualizations and analysis were done using the following R packages: tidyverse, dendextend, cluster, vegan and pheatmap. Please see the last paragraph in the methodology section and references section for further details.

For manuscripts utilizing custom algorithms or software that are central to the research but not yet described in published literature, software must be made available to editors and reviewers. We strongly encourage code deposition in a community repository (e.g. GitHub). See the Nature Research guidelines for submitting code & software for further information.

Data

Policy information about availability of data

All manuscripts must include a data availability statement. This statement should provide the following information, where applicable:

- Accession codes, unique identifiers, or web links for publicly available datasets
- A list of figures that have associated raw data
- A description of any restrictions on data availability

The dataset created has been made available as a supplementary dataset.

Field-specific reporting

Please select the one below that is the best fit for your research. If you are not sure, read the appropriate sections before making your selection.

☐ Life sciences ☐ Behavioural & social sciences ☒ Ecological, evolutionary & environmental sciences

For a reference copy of the document with all sections, see nature.com/documents/nr-reporting-summary-flat.pdf

Ecological, evolutionary & environmental sciences study design

All studies must disclose on these points even when the disclosure is negative.

Study description	A systematic review on trade-off analysis within agriculture.
Research sample	The research sample consisted of all peer-reviewed articles from the Web of Science database that corresponded to our search string.
Sampling strategy	We used the following search string: “ALL=agricultur* AND ("trade off* analysis" OR "trade-off* analysis" OR "tradeoff* analysis")” in the Web of Science (on 14/09/2021) to identify peer-reviewed articles in English conducting TOA. We found 153 articles with publication dates spanning from 1993 to 2021.
Data collection	Criteria to evaluate for each article were defined prior to the review. TB read and evaluated all articles based on criteria. (See meta data in data file). Decisions made during evaluation were also logged in the 'Rules' tab in the data file.
Timing and spatial scale	Data collection took place from 15-09-2021 up till 31-01-2022, the date at which all articles had been read and logged.
Data exclusions	Review- and opinion papers were considered off-topic and were excluded from the search results. Furthermore, methodological papers that did not involve a case-study were also excluded, leading to a total sample of 119 articles.
Reproducibility	Reproducibility has been facilitated through making the data file accessible together with the notes and decision-making in the data file, if required we can also publish the R code used to analyze the data and visualize results.
Randomization	After downloading all the articles, they were evaluated in alphabetical order based on author names.
Blinding	We did not consider blinding applicable to our study. One way in which it could have been would be to blind the authors and their affiliation. However, we also wanted to use this information to investigate geographic trends between the author's affiliation and the location of the case-study (see suppl. material).

Did the study involve field work? ☐ Yes ☒ No

Reporting for specific materials, systems and methods

We require information from authors about some types of materials, experimental systems and methods used in many studies. Here, indicate whether each material, system or method listed is relevant to your study. If you are not sure if a list item applies to your research, read the appropriate section before selecting a response.

Materials & experimental systems

n/a	Involved in the study
☒	☐ Antibodies
☒	☐ Eukaryotic cell lines
☒	☐ Palaeontology and archaeology
☒	☐ Animals and other organisms
☒	☐ Human research participants
☒	☐ Clinical data
☒	☐ Dual use research of concern

Methods

n/a	Involved in the study
☒	☐ ChIP-seq
☒	☐ Flow cytometry
☒	☐ MRI-based neuroimaging
